# Effects of *Aronia melanocarpa* Tannins on Oxidative Stress and Immune Dysfunction

**DOI:** 10.3390/molecules30224338

**Published:** 2025-11-08

**Authors:** Kseniya Bushmeleva, Alexandra Vyshtakalyuk, Dmitriy Terenzhev, Timur Belov, Kamila Kazimova, Vladimir Zobov

**Affiliations:** A.E. Arbuzov Institute of Organic and Physical Chemistry, Kazan Scientific Center, Russian Academy of Sciences, Arbuzov str. 8, Kazan 420088, Russia; alex.vysh@mail.ru (A.V.); vz30608@mail.ru (V.Z.)

**Keywords:** extraction, cyclophosphamide-induced toxicity, natural antioxidants, phytochemical characterization, membrane protection, neutrophil oxidative metabolism

## Abstract

Natural polyphenols, particularly tannins, are of interest due to their complex composition and multi-target biological activities. A highly purified tannin fraction was isolated from *Aronia melanocarpa* fruits, and its composition was characterized by HPLC-MS and IR spectroscopy. The Aronia tannin fraction exhibited comprehensive antioxidant properties, demonstrating superior DPPH radical scavenging activity compared to quercetin and a membrane-protective effect exceeding reference antioxidants. In vivo, Aronia tannins showed a delayed but potent antioxidant effect against cyclophosphamide (CP)-induced oxidative stress, significantly reducing malondialdehyde (MDA) levels, with the maximum effect observed at days 14–21. The immunomodulatory effect involved a complex regulation of the phagocytic system: selective activation of the monocytic arm with simultaneous modulation of neutrophilic activity. Crucially, a high phagocytic completion rate was maintained, indicating support for both bacterial uptake and intracellular killing. Tannins accelerated recovery post-CP, restoring leukocyte and platelet counts. Modulation of neutrophil oxidative metabolism, measured by chemiluminescence, indicates an ability to balance defense activation with prevention of excessive oxidative stress. These findings confirm the potential of the *Aronia melanocarpa* tannin fraction for correcting oxidative stress and immune dysfunction.

## 1. Introduction

Despite its widespread use as an alkylating agent, cyclophosphamide induces a spectrum of pathological effects including myelosuppression, oxidative stress, immune dysfunction, and organ toxicity. Current corrective agents often demonstrate a narrow therapeutic focus and fail to prevent long-term therapy complications. The fundamental challenge lies in balancing aggressive cytostatic therapy with preserving patients’ quality of life, necessitating novel pharmacological approaches. Lipid peroxidation-mediated membrane damage and hematopoietic disruption significantly limit treatment efficacy and compromise patient wellbeing. Existing corrective agents frequently prove inadequate or introduce additional adverse effects. Consequently, developing novel agents that provide comprehensive protection against cytostatic toxicity while maintaining favorable safety profiles represents a crucial research priority.

A promising strategy involves utilizing natural polyphenol complexes, which may exhibit synergistic effects through multi-target actions on various components of the pathological process [1]. In this context, black chokeberry (*Aronia melanocarpa* (Michx.) Elliott) is of particular interest. It is a deciduous shrub belonging to the *Rosaceae* family, widely cultivated for its fruits, which are distinguished by an exceptionally high content of bioactive polyphenols [2]. The chemical composition of Aronia berries is unique, encompassing anthocyanins, predominantly cyanidin-3-galactoside, phenolic acids such as chlorogenic and neochlorogenic acid, and, most importantly for this study, significant amounts of proanthocyanidins—condensed tannins with varying degrees of polymerization [3]. It is this complex composition that underlies the pronounced antioxidant, membrane-protective, and immunomodulatory activities of aronia extracts, finding applications in functional foods and pharmacology [4,5].

It should be noted that tannins, which include the proanthocyanidins from Aronia, constitute a broad class of phenolic compounds traditionally subdivided into two main groups: hydrolyzable tannins (e.g., tannic acid) and condensed tannins (proanthocyanidins) [6]. The biological activity of tannins, including their antioxidant, anti-inflammatory, and immunomodulatory effects, is largely attributed to their ability to interact with proteins and other macromolecules [7]. Furthermore, the complex, multi-component nature of an isolated tannin fraction, as opposed to a single, pure compound, may lead to synergistic effects, resulting in a more balanced and multi-targeted physiological impact [8]. However, the properties of specific tannin fractions and their mechanisms of action under chemotherapy conditions remain poorly explored compared to individual tannins or synthetic antioxidants.

Therefore, this study aimed to comprehensively investigate the antioxidant, membrane-protective, immunomodulatory, and hemoprotective properties of an *Aronia melanocarpa* tannin fraction, compared to tannic acid and reference antioxidants (ascorbic acid, Trolox, and quercetin), in a cyclophosphamide-induced immunosuppression model. The findings will provide a scientific rationale for the advantages of using complex tannin fractions over individual compounds to treat pathologies of various etiologies and develop novel approaches to enhance the efficacy and safety of therapies for oxidative stress-related diseases.

## 2. Results

### 2.1. Chemical Composition of Extracts

The identification of major phytochemical groups involved determining the total content of phenols, flavonoids, anthocyanins, sugars, and tannins in 70% ethanolic extracts of *Aronia melanocarpa* fruits from the ‘Chernaya Zhemchuzhina’ (Black Pearl) and ‘Chernookaya’ cultivars, as presented in Table 1.

According to Table 1, the highest total tannin content was observed in the 70% ethanolic extract of the ‘Chernookaya’ cultivar berries (27.3% higher than in the ‘Black Pearl’ cultivar). The 70% ethanolic extract of the ‘Chernookaya’ berries also exhibited higher phenolic and flavonoid contents (24.7% and 19.13% higher, respectively, compared to the ‘Black Pearl’ cultivar). In contrast, the anthocyanin and sugar contents were significantly lower (by 41.99% and 29.43%, respectively).

The elevated tannin content coupled with lower anthocyanin and sugar levels in the ‘Chernookaya’ berry extract justified its selection for further studies aimed at obtaining tannin-enriched extracts.

For the *Aronia melanocarpa* ‘Chernookaya’ cultivar, optimal extraction solvents were identified to maximize tannin yield: 70% acetone, 75% methanol, and a 50% ethanol solution with 0.22% sodium hydroxide (pH = 8.0). The results for the total content of sugars, phenolic compounds, flavonoids, anthocyanins, and tannins are presented in Table 2.

As shown in Table 2, the highest tannin content was achieved using a 50% ethanol solution with 0.22% sodium hydroxide as the extraction solvent, with values 13.41% and 30.47% higher than those obtained with 75% methanol and 70% acetone, respectively.

The data demonstrate significant solvent-dependent variations in the extraction efficiency of major phytochemical groups. Phenolic content was lowest when using 70% acetone, showing a reduction of 18.66% and 42.2% compared to the other two solvents. Flavonoid and anthocyanin levels were minimal with 75% methanol, reaching only 1.13 mg Rut/g DE and 1.12 mg/g DE, respectively.

Total sugar content was highest in the extract obtained with the 50% ethanol solution containing 0.22% sodium hydroxide, which is characteristic of a high-polarity solvent system. The sugar content with this solvent exceeded that of the other two extracts by factors of 2.17 and 3.78.

For subsequent tannin fraction isolation and purification, the ‘Chernookaya’ berry extract obtained with the 50% ethanol solution containing 0.22% sodium hydroxide was selected. Following gel filtration chromatography on Sephadex LH-20, the maximum tannin content was observed in fractions 9–11, showing a 9.97-fold increase compared to the crude extract. Fractions 3–6 and 14–17 demonstrated 3.4-fold and 2.9-fold higher tannin content, respectively.

Fraction 9–11, obtained after purification with Sephadex LH-20 gel filtration resin, exhibited significantly reduced sugar content compared to the initial 50% ethanolic extract with 0.22% sodium hydroxide (a 99.62% decrease). Anthocyanin and flavonoid contents were also substantially reduced by 93.92% and 91.10%, respectively. Fractions 3–6 and 14–17 showed comparatively higher sugar retention, with decreases of 66.31% and 83.95% relative to the initial extract. Notably, anthocyanins were not detected spectrophotometrically in fraction 14–17.

Residual sugar, anthocyanin and flavonoid content in fraction 9–11 was confirmed by HPLC-MS analysis based on retention time, elution order, spectroscopic characteristics, and fragmentation patterns. The results are presented in Table 3. Two major anthocyanin peaks were identified in this fraction: cyanidin-3-O-galactoside and cyanidin-3-O-arabinoside. In contrast, the initial 50% ethanolic extract with 0.22% sodium hydroxide contained two additional anthocyanins: cyanidin-3-O-glucoside and cyanidin-3-O-xyloside, consistent with previous findings [9].

The identified anthocyanins accounted for 97.98% of the total anthocyanin content in tannin fraction 9–11 and 98.82% in the 50% ethanolic extract with 0.22% sodium hydroxide (Figures S1 and S2). As shown in Table 4, the content of cyanidin-3-O-galactoside (Cy-3-Gal) in fraction 9–11 decreased by 90.3% following column chromatography, while cyanidin-3-O-arabinoside (Cy-3-Ara) content decreased by 93.81%.

HPLC-MS analysis also enabled quantification of flavonol compounds in both the 50% ethanolic extract with 0.22% sodium hydroxide and tannin fraction 9–11 from *A. melanocarpa* ‘Chernookaya’. These flavonols, belonging to the flavonoid class, were identified in both aglycone and glycosylated forms, as presented in Table 4.

The detected flavonol content accounted for 96.99% of the total flavonoid content in the 50% ethanolic extract with 0.22% sodium hydroxide, and 97.62% in tannin fractions 9–11 (Figure S3). We suggest that the remaining unidentified compounds in the dry extract belonged to the class of flavonic acids and were below the detection limit [10].

The identified sugars in the analyzed samples were quantified using standard solutions of fructose, glucose, sorbitol, sucrose, and maltose (Figure S4a). The analytical results are presented in Table 5. Four sugar peaks were identified in the initial 50% ethanolic extract of Aronia with 0.22% sodium hydroxide, corresponding to fructose, glucose, sorbitol, and sucrose, which aligns with findings from previous studies [11,12].

The detected sugar content accounted for 95.91% of the total sugar content in the 50% ethanolic extract with 0.22% sodium hydroxide, and 97.12% in tannin fraction 9–11 (Figure S4b,c). Sucrose was not identified in fraction 9–11.

**Figure 1:**
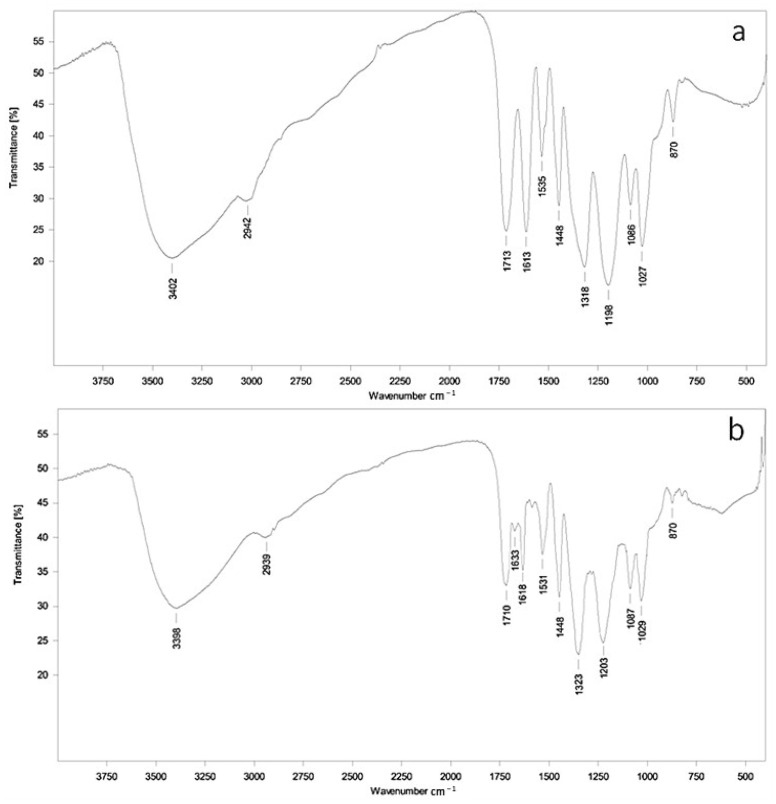
IR spectra. (**a**) Tannic acid; (**b**) tannin fraction 9–11.

Qualitative information about the functional group composition of tannic acid and the tannin fraction was obtained using Fourier-transform infrared (FTIR) spectroscopy (Figure 1). The FTIR spectra of both samples (Figure 1a,b) exhibit intense absorption bands, with the tannic acid and *Aronia melanocarpa* ‘Chernookaya’ tannin fraction spectra demonstrating similar profiles.

The tannin fraction spectrum differed from the tannic acid spectrum by lower intensity of absorption bands. The first distinctive peak in the 3398–3402 cm^−1^ region indicates a high degree of OH-group involvement in hydrogen bonding, while the broad peak in the 2939–2942 cm^−1^ region is associated with C-H bond stretching vibrations in CH_3_ and CH_2_ groups. The peak at 1710–1713 cm^−1^ corresponds to C=O bond stretching of carboxyl groups, and the 1613–1618 cm^−1^ peaks indicate characteristic aromatic ring stretching vibrations typical of tannins [13].

The registered FTIR spectra exhibit absorption bands at 1531 and 1535 cm^−1^, characteristic of C-C stretching vibrations in non-condensed aromatic compounds. The absorption band at 1448 cm^−1^ represents aromatic ring stretching vibrations, while bands at 1318 and 1323 cm^−1^ correspond to vibrations associated with hydroxyl groups. Intense vibration bands at 1198 and 1203 cm^−1^ are typical of hydroxyl groups in phenolic fragments; bands at 1086–1087 cm^−1^ and 1027–1029 cm^−1^ represent in-plane C-H deformation vibrations of aromatic rings. A weakly expressed absorption band at 870 cm^−1^ indicates stretching vibrations of symmetric C-O-C bonds and C-O bonds in the ring structure of flavonoid-based tannins.

The tannin fraction spectrum additionally shows a peak at 1663 cm^−1^, suggesting C=O stretching vibrations, whose weak intensity indicates the presence of minor flavonoid content.

While flavonols and anthocyanins are known to contribute to the antioxidant properties of plant extracts [14], we suggest that the residual amounts of anthocyanins and flavonoids in tannin fraction 9–11 are unlikely to contribute significantly to the overall antioxidant activity.

### 2.2. DPPH Radical Scavenging Activity of the Tested Compounds

DPPH analysis successfully revealed the antioxidant potential of the selected compounds (Table 6). Ascorbic acid demonstrated the ability to reduce the chromogen radical at the lowest concentration, indicating strong antioxidant properties, which is confirmed by its lowest EC_50_ value (0.010 mg/mL).

The flavonoid-based antioxidant quercetin demonstrated 14.9-fold lower antioxidant activity (EC_50_ = 0.149 mg/mL) in this assay, which may be attributed to its limited solubility in the analysis medium. Tannic acid showed 1.68-fold greater DPPH radical inhibition efficacy compared to the tannin isolated from Aronia fruits.

The molecular structure of tannins, characterized by multiple hydroxyl groups, enables them to function as potent free radical scavengers. Ascorbic acid and tannic acid demonstrated the most rapid suppression of DPPH radicals.

### 2.3. Chemiluminescence Activity of the Tested Compounds

**Figure 2:**
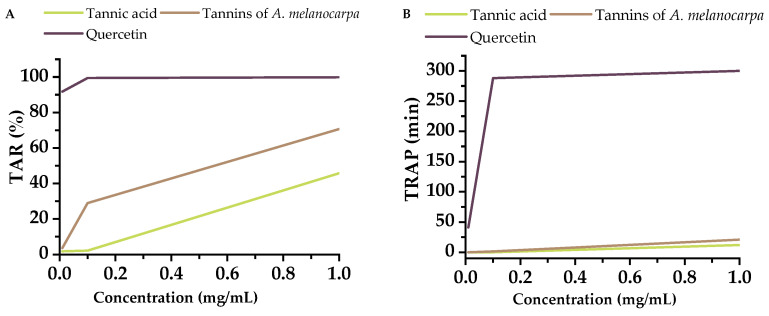
Analysis of antioxidant activity by chemiluminescence assay: (**A**) TAR (Total Antioxidant Reactivity) and (**B**) TRAP (Total Radical-trapping Antioxidant Parameter) results.

In our study, quercetin demonstrated exceptionally high TRAP (Total Radical-Trapping Antioxidant Parameter) values at all tested concentrations, showing superior capacity to delay free radical-induced oxidation compared to tannins (Figure 2). Specifically, at a concentration of 1 mg/mL, quercetin was 25.1 times more effective than tannic acid and 14.5 times more effective than the Aronia tannin fraction in delaying oxidation.

The TAR value of tannic acid was 2.2 times lower than that of quercetin at 1 mg/mL concentration. The TAR value of the Aronia tannin fraction was 1.41 times lower than that of quercetin and 1.54 times higher than that of commercial tannin at 1 mg/mL concentration.

Differences between tannic acid and Aronia-derived tannin samples were minimal and showed significant reduction in reactivity at concentrations of 0.1 mg/mL and 0.01 mg/mL.

The lower activity of tannins may be attributed to their complex polymeric structures or interactions with the solvent system.

Quercetin performs more effectively in systems with prolonged oxidative stress (TRAP) than in rapid radical tests (DPPH).

### 2.4. Effect of Tannins on Osmotic and Peroxide-Induced Hemolysis

**Figure 3:**
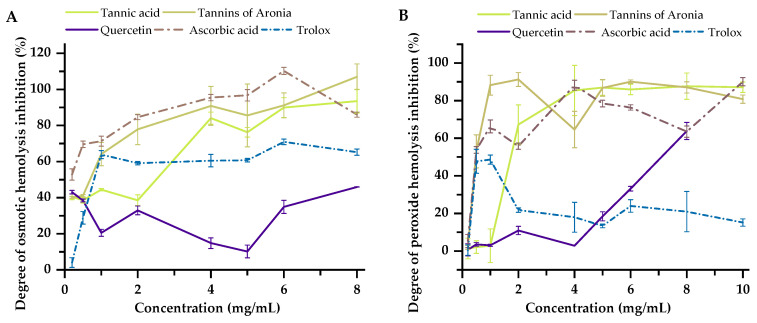
Concentration-dependent inhibition of hemolysis in rat erythrocytes: (**A**) osmotic hemolysis induced by hypotonic shock; (**B**) peroxidative hemolysis induced by Fenton reaction.

The investigation of compound effects on erythrocyte osmotic hemolysis is crucial for assessing compound-induced cellular stress. This study examined the in vitro protective effects of potential anti-inflammatory molecules—Aronia tannins, tannic acid, quercetin, ascorbic acid, and Trolox—on rat erythrocyte osmotic fragility in a hypotonic environment (Figure 3A).

The protective effect of tannins is believed to stem from their interaction with erythrocyte membranes, potentially stabilizing them against osmotic stress. Both *Aronia melanocarpa* tannin and commercial tannic acid demonstrated concentration-dependent inhibition of osmotic hemolysis in rat erythrocytes—with increasing concentrations from 0.2 to 8 mg/mL, the degree of erythrocyte membrane protection increased. At concentrations of 4 mg/mL and above, Aronia tannin and commercial tannic acid exhibited significant protective effects—hemolysis inhibition exceeded 80%, surpassing the effects of quercetin (by 6.14-fold and 5.68-fold, respectively) and Trolox (by 1.5-fold and 1.39-fold, respectively). At a 1 mg/mL concentration, *Aronia melanocarpa* tannin provided a 1.44-fold greater membrane-protective effect, indicating higher activity compared to tannic acid. Similar degrees of hemolysis inhibition were observed for both compounds at low concentrations (0.2 mg/mL and 0.5 mg/mL).

Ascorbic acid proved to be the most potent protective agent against osmotic hemolysis across all tested concentrations. Its membrane-protective activity increased with concentration, reaching a maximum hemolysis inhibition of 110.26% at 6 mg/mL. Quercetin exhibited a non-monotonic response, providing some protection at lower concentrations (0.2 mg/mL and 0.5 mg/mL) but showing reduced efficacy at higher concentrations (1–6 mg/mL), potentially due to its limited solubility in physiological media. Trolox demonstrated moderate hemolysis protection with consistent concentration-dependent inhibition up to 8 mg/mL.

Analysis of compound effects on rat erythrocyte hemolysis induced by hydroxyl radicals (Fenton reaction) revealed a concentration-dependent response for tannic acid (Figure 3B). At concentrations ≥ 2 mg/mL, tannic acid provided substantial protection, with inhibition increasing from 67.29% at 2 mg/mL to 87.12% at 10 mg/mL.

Aronia tannin exhibited enhanced membrane-protective activity at concentrations ≥ 0.5 mg/mL, reducing hemolysis by up to 53%—representing a 50.9% greater effect compared to tannic acid at equivalent concentrations. Overall, the protective effects of both tannins were comparable to those of ascorbic acid on erythrocyte membranes.

Ascorbic acid demonstrated concentration-dependent protection against free radical-induced hemolysis, with maximum inhibition (90.08%) observed at 10 mg/mL. Trolox showed significantly lower free radical scavenging capacity on erythrocyte membranes compared to the tested tannins. The membrane-protective effects of quercetin and Trolox were inferior to ascorbic acid—at 2 mg/mL, their efficacy was lower by 44.8% and 34%, respectively. Trolox efficacy varied within a 19% range at concentrations ≥ 2 mg/mL, while quercetin showed increasing protection at concentrations ≥ 4 mg/mL, though never surpassing the protective effect of ascorbic acid.

### 2.5. Analysis of Tannin Cytotoxicity

**Figure 4:**
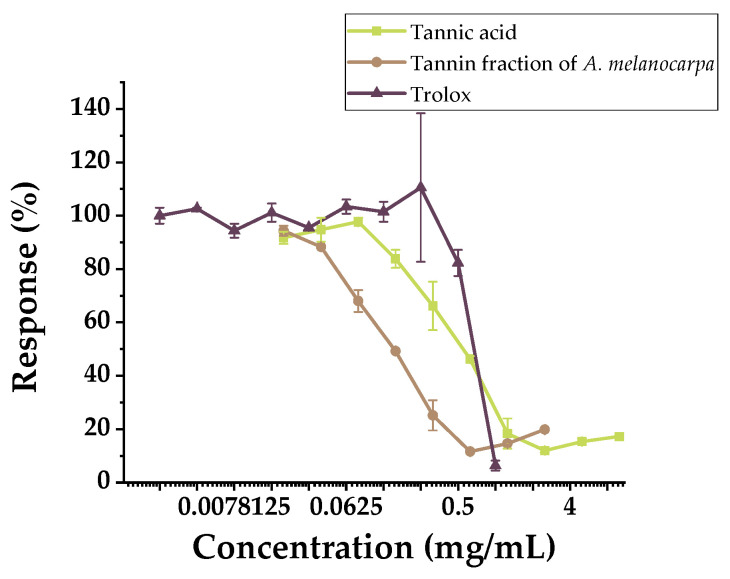
Effect of investigated compounds on Chang liver cell viability. Green line—commercial tannins; brown line—*A. melanocarpa* tannins; purple line—Trolox. Calculations performed using the online tool: https://www.aatbio.com/tools/ic50-calculator/, accessed on 23 September 2025.

The cytotoxicity (IC_50_) of the investigated compounds was determined, with results presented in Figure 4.

Over the broad concentration range tested (0.0195–10 mg/mL), the compounds exhibited cytotoxicity against the conditionally normal Chang liver cell line. The IC_50_ of Aronia tannin was 0.1204 mg/mL (120.4 mg/L). The high biological activity of tannins is often attributed to the same fundamental property: their capacity to form strong complexes with proteins and biomembranes and to inhibit cytochrome P450 activity [15]. In short-term experiments (hemolysis), this property stabilizes the erythrocyte membrane, protecting it from external stress. However, during prolonged exposure to metabolically active cells, this same property disrupts the function of membrane receptors, enzymes, and mitochondria, leading to suppressed metabolism and cell death [16].

The IC_50_ of commercial tannic acid was 0.4516 mg/mL (451.6 mg/L), making it 3.75 times less cytotoxic than Aronia tannin. This difference is explained by molecular composition (degree of polymerization and monomer profile), purification level, and the manifestation of pro-oxidant properties in liver cells due to its high oxygen content [17].

The IC_50_ of Trolox was 0.611 mg/mL (611 mg/L). Trolox’s mechanism of action involves quenching free radicals in the aqueous phase without strong interaction with hepatic enzyme systems. It is metabolically inert and does not significantly negatively impact hepatocyte viability within the concentration range used [18].

### 2.6. Analysis of Lipid Peroxidation

**Figure 5:**
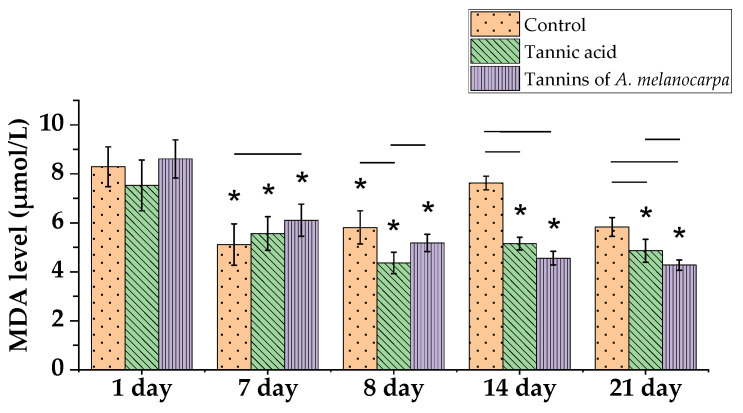
Malondialdehyde (MDA) level in rat peripheral blood. * (*p* < 0.05)—Significant difference compared to day 1 within the same group. – (*p* < 0.05)—Significant difference between treatment groups.

MDA (malondialdehyde) is a terminal product of polyunsaturated fatty acid peroxidation in cell membranes resulting from reactive oxygen species (ROS) activity. The results of MDA level analysis demonstrate a pronounced protective effect of tannins against cyclophosphamide (CP)-induced oxidative stress (Figure 5).

Cyclophosphamide-induced pronounced oxidative stress—in the control group, MDA levels increased 1.3-fold compared to day 8 data, with a peak elevation to 7.63 μmol/L observed on day 14 (*p* < 0.05). Tannic acid exhibited a rapid protective effect—MDA levels decreased 1.2-fold relative to day 7 within 24 h after CP administration (day 8). In contrast, Aronia tannins demonstrated a delayed but more potent effect—on day 8, a 1.2-fold reduction in MDA was observed, which persisted throughout the experiment. The maximum reduction in MDA levels occurred by day 21, reaching 4.28 μmol/L.

Both tannin types effectively suppressed the oxidative stress peak—on day 8, MDA levels in tannin-treated groups were 1.3-fold lower than in the control. By day 14, MDA levels in the tannic acid and Aronia tannin groups were significantly lower than control values by 1.5-fold and 1.7-fold, respectively (*p* < 0.05). Similarly, on day 21, levels remained lower than control values by 1.2-fold and 1.4-fold, respectively (*p* < 0.05). Notably, MDA levels in the tannic acid group were 1.14-fold higher than in the Aronia tannin group (*p* < 0.05).

### 2.7. Analysis of Rat Peripheral Blood Hematological Parameters

**Figure 6:**
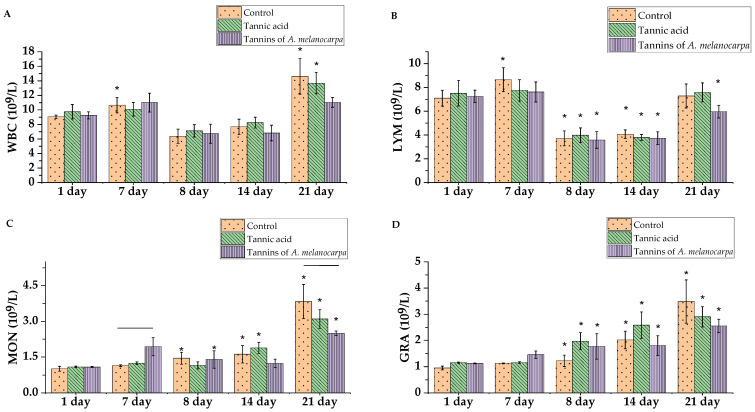
Leukocyte profile. (**A**) White blood cell count (WBC); (**B**) Lymphocyte count (LYM); (**C**) Monocyte count (MON); (**D**) Neutrophilic granulocyte count (GRA). * (*p* < 0.05)—Significant difference compared to Day 1 within the same group. – (*p* < 0.05)—Significant difference between treatment groups.

Hematological analysis revealed that in the preventive regimen, the number of monocytes increased in the group of rats administered the Aronia tannin fraction for 7 days, being 1.69-fold higher than in the control group (Figure 6C) (*p* < 0.05). Additionally, in the preventive regimen, the lymphocyte count in the control group increased 1.22-fold (*p* < 0.05), which may represent a stress response (Figure 6B).

In response to CP administration, all rat groups developed leukopenia and lymphopenia due to suppressed proliferation of lymphoid and myeloid progenitor cells in the bone marrow (Figure 6A). White blood cell counts decreased, primarily lymphocytes, while monocyte and neutrophil granulocyte counts increased (*p* < 0.05). This effect persisted for an additional 7 days.

By experimental day 21, an increase in white blood cell count was observed, indicating myelopoiesis recovery. In the tannic acid group, a gradual and significant increase in granulocyte count was noted: 1.7-fold after CP administration, 2.24-fold on day 14, and 2.52-fold on day 21 compared to baseline day 1 values (Figure 6D) (*p* < 0.05). In the Aronia tannin fraction group, granulocyte increase was less pronounced: 1.58-fold after CP, 1.61-fold on day 14, and 2.28-fold on day 21, respectively (*p* < 0.05).

The control group also showed increased granulocyte counts: 1.27-fold after CP, 2.13-fold on day 14, and 3.66-fold on day 21 (*p* < 0.05). Notably, on day 21, granulocyte counts in the control group exceeded those in other groups by 1.2-fold and 1.36-fold. Similarly, monocyte counts on day 21 were 1.24-fold and 1.53-fold higher than in the tannic acid and Aronia tannin groups, respectively (*p* < 0.05).

**Figure 7:**
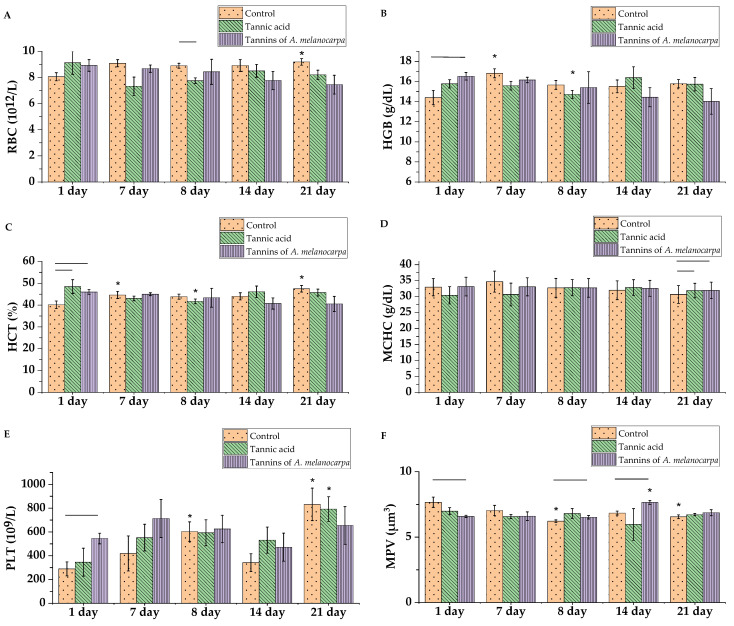
Erythrocyte and platelet profile. (**A**) Red blood cell count (RBC); (**B**) Hemoglobin concentration (HGB), (**C**) Hematocrit (HCT); (**D**) Mean corpuscular hemoglobin concentration (MCHC); (**E**) Platelet count (PLT); (**F**) Mean platelet volume (MPV). * (*p* < 0.05)—Significant difference compared to day 1 within the same group. – (*p* < 0.05)—Significant difference between treatment groups.

The erythrocyte profile showed minimal changes under tannic acid treatment (Figure 7). Hemoglobin concentration spontaneously increased 1.17-fold in the control group on day 7 (Figure 7B) (*p* < 0.05). Red blood cell count (Figure 7A) and hematocrit (Figure 7C) decreased 1.25-fold and 1.13-fold, respectively, on day 7 after tannic acid administration (*p* < 0.05). Following CP administration, RBC count in the tannic acid group remained 1.14-fold lower than in controls (*p* < 0.05). Hemoglobin concentration and hematocrit after tannic acid treatment were 1.08-fold and 1.16-fold lower, respectively, compared to day 1 values.

On day 21 of the experiment, RBC count and hematocrit in the control group were higher than in the experimental groups—these parameters increased 1.14-fold and 1.18-fold relative to baseline values, respectively (*p* < 0.05). MCHC—indicating erythrocyte hemoglobin saturation—was 1.04-fold lower in the control group compared to the tannic acid and Aronia tannin fraction groups on day 21 (Figure 7D) (*p* < 0.05).

Analysis of platelet parameters revealed decreased MPV in control group rats 24 h after CP administration (1.24-fold lower than on day 1 and 1.05-fold lower than in the Aronia tannin fraction group, *p* < 0.05), which is characteristic of bone marrow suppression (Figure 7F).

Seven days after CP administration, platelet count in the control group decreased 1.76-fold compared to day 8 values, while decreasing 1.12-fold in the tannic acid group and 1.32-fold in the Aronia tannin fraction group (Figure 7E). Mean platelet volume increased 1.17-fold relative to baseline values in the Aronia tannin fraction group and was 1.13-fold higher than in the control group (*p* < 0.05), indicating the release of large young platelets during recovery.

By day 21, platelet count and mean platelet volume increased in both the control group and the tannic acid group (*p* < 0.05).

### 2.8. Analysis of Leukocyte Phagocytic Activity

**Figure 8:**
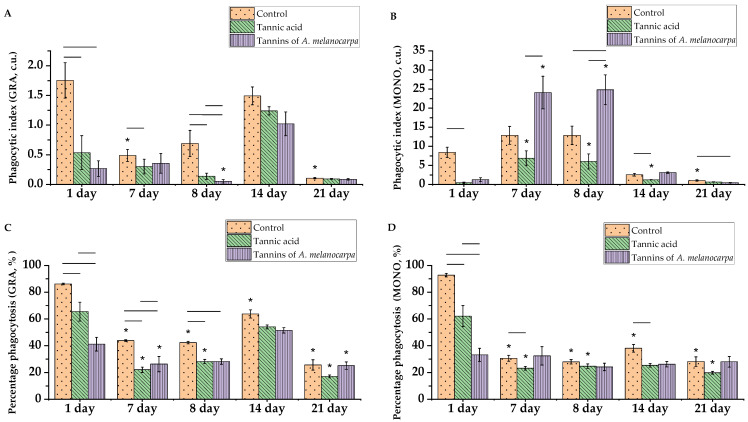

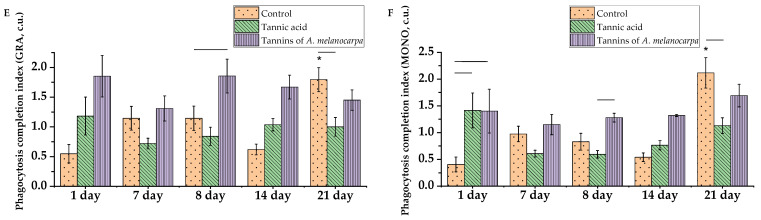
Phagocytic activity parameters of granulocytes and monocytes in rat peripheral blood. Phagocytic index (panels (**A**,**B**), c.u.), phagocytosis percentage (panels (**C**,**D**), %), and phagocytic completion index (panels (**E**,**F**), c.u.). * (*p* < 0.05)—significant difference compared to day 1 within the group. – (*p* < 0.05)—significant difference between groups.

Phagocytosis analysis revealed that the average number of bacterial particles ingested by one active neutrophil (phagocytic number, PN, of neutrophils) in the preventive regimen decreased 1.8-fold in the control group and 4.3-fold in the group receiving the Aronia tannin fraction (Figure S5A) (*p* < 0.05). On day 7 of tannic acid administration, the average number of granulocytes decreased 1.6-fold (*p* < 0.05). The average number of bacterial particles per monocyte (phagocytic index, PI) increased 15.6-fold in the tannic acid group and 19.2-fold in the Aronia tannin fraction group by day 7 (Figure 8A). The phagocytic number (PN) of monocytes increased in all groups (Figure S5B). (*p* < 0.05). The phagocytic index (PI) of monocytes in the Aronia tannin fraction group was 3.5-fold higher compared to the tannic acid group (Figure 8B) (*p* < 0.05).

The percentage phagocytosis (PP), representing the proportion of active granulocytes (Figure 8C) and monocytes (Figure 8D), spontaneously decreased by 2-fold and 3-fold, respectively, in the control group by day 7, and by 3-fold and 2.7-fold in the group receiving the tannic acid solution (*p* < 0.05). In the group administered the Aronia tannin fraction, the granulocyte PP decreased 1.5-fold, while the monocyte PP remained unchanged. On day 7, the proportion of active neutrophils in the control was higher than in the experimental groups, and the proportion of active monocytes was 1.3-fold higher in the control group compared to the tannic acid group (*p* < 0.05).

Thus, while the control group exhibited natural phagocytosis destabilization, both tannin forms suppressed excessive monocyte activation, maintained neutrophil activity, and reduced parameter variability. According to the literature, ellagitannins modulate NF-κB and reduce spontaneous monocyte activation in controls without suppressing it during actual infection [19].

On day 8 post-immunosuppression induction, the monocyte phagocytic index (PI) increased 13.7-fold and 19.8-fold in the tannic acid and Aronia tannin fraction groups, respectively, with monocyte phagocytic number (PN) increasing 22.5-fold and 148-fold relative to day 1 (*p* < 0.05). The number of bacterial particles in monocytes was 1.9-fold and 4.2-fold higher than in the control and tannic acid groups, respectively (*p* < 0.05). Conversely, neutrophil bacterial particle count was 3.2-fold and 5.8-fold higher in the control compared to the tannic acid and Aronia tannin groups (*p* < 0.05).

Following CP exposure, neutrophil and monocyte PP showed no significant differences relative to pre-CP values but were significantly lower compared to day 1, except for monocyte PP in the Aronia tannin fraction group. Similar to day 7, the proportion of active neutrophils remained higher in the control on day 8.

On day 8, the phagocytosis completion index (PCI) for granulocytes was 1.62-fold higher in the Aronia tannin group compared to controls (Figure 8E), while monocyte PCI was 1.54-fold higher than in the tannic acid group (Figure 8F) (*p* < 0.05).

By day 14, the control group showed increased bacterial particles in neutrophils: PI and PC increased 2.2-fold, but decreased in monocytes by 5.1-fold and 6.5-fold (*p* < 0.05). The tannic acid group similarly exhibited increased granulocyte PI and PN (8.9-fold and 4.7-fold) but decreased monocyte PI and PN (4.9-fold and 3.9-fold) (*p* < 0.05). Monocyte PI was 2.1-fold lower in the tannic acid group than in controls (*p* < 0.05). The Aronia tannin group showed increased granulocyte PI and PN (20.4-fold and 7.2-fold) but decreased monocyte PI and PN (8-fold and 5.4-fold) (*p* < 0.05).

Granulocyte and monocyte PP increased 1.5-fold relative to days 7–8 in controls (*p* < 0.05). The tannic acid group showed 2.5-fold and 1.9-fold increases in granulocyte PR relative to days 7 and 8, respectively (*p* < 0.05). However, monocyte PR was 1.5-fold lower in the tannic acid group than in controls (*p* < 0.05). Granulocyte PR increased 1.8-fold by day 14 in the Aronia tannin group (*p* < 0.05).

A decreasing trend in neutrophil and granulocyte PCI was observed in controls, while experimental groups showed increased PCI.

By day 21, significant reductions in bacterial particles occurred: neutrophil PI and PC decreased 13.5-fold and 4.4-fold in controls, with monocyte PI decreasing 2.5-fold (*p* < 0.05). Similar reductions occurred in the tannic acid group (neutrophil PI/PN: 13.8-fold/3.8-fold; monocyte PI: 1.8-fold) and the Aronia tannin group (neutrophil PI/PN: 11.3-fold/2.2-fold; monocyte PI: 6.7-fold) (*p* < 0.05).

Granulocyte and monocyte PP decreased 2.5-fold and 1.4-fold relative to day 14 in controls, while decreasing 3.2-fold in the tannic acid group and 2-fold in the Aronia tannin group (*p* < 0.05). By day 21, granulocyte PCI increased 2.9-fold relative to day 14 in controls.

### 2.9. Analysis of Spontaneous and Activated Chemiluminescent Activity of Neutrophils

**Figure 9:**
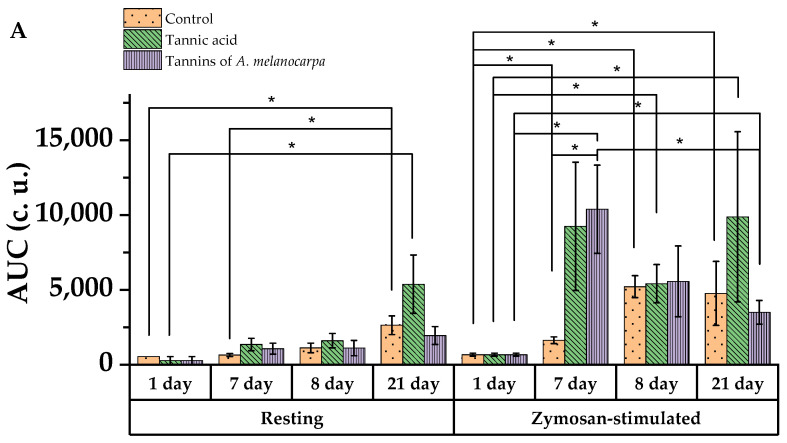

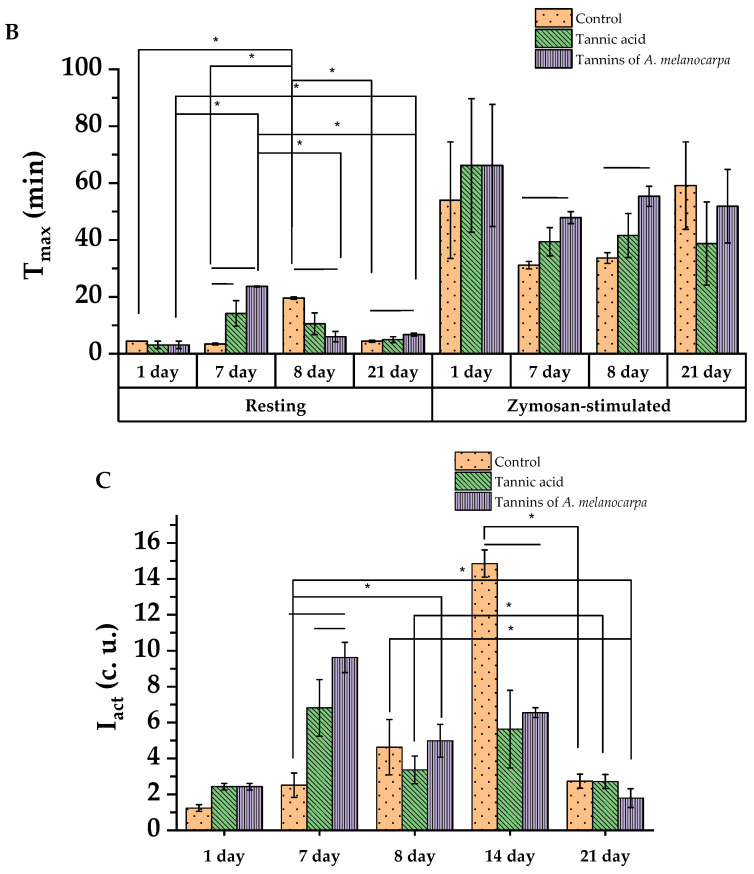
Parameters of spontaneous and zymosan-activated chemiluminescence of rat neutrophils: (**A**) area under the luminescence curve (AUC, c.u.), (**B**) time to peak chemiluminescence (T_max_, min), (**C**) activation index (I_act_, c.u.). * (*p* < 0.05)—Significant difference compared to day 1 within the same group. – (*p* < 0.05)—Significant difference between treatment groups.

Chemiluminescence analysis of neutrophil activity is a highly sensitive method for assessing the functional state of these cells, particularly their ability to generate reactive oxygen species (ROS). This approach is based on detecting light emission during oxidative reactions upon neutrophil activation. In the preventive regimen, an increase in chemiluminescence intensity (I_max_) was observed for both background and activated levels across all experimental groups: 4.05-fold and 7.55-fold in the control group, 9.44-fold and 13.6-fold in the tannic acid group, and 5.6-fold and 12-fold in the Aronia tannin fraction group (Figure S6A) (*p* < 0.05). The integral parameter of total ROS production—the area under the chemiluminescence curve (AUC)—increased 2.4-fold in the control group and 15.3-fold in the Aronia tannin fraction group (Figure 9A) (*p* < 0.05). Notably, in the latter group, both AUC and time to peak background chemiluminescence (T_max_) (Figure 9B) were significantly higher than in controls: 6.33-fold and 1.53-fold, respectively (*p* < 0.05). Interestingly, spontaneous T_max_ in the control group was lower compared to both tannic acid and Aronia tannin fraction groups, while the chemiluminescence curve slope (Slope) was steeper in the control group (Figure S6B) (*p* < 0.05).

Following immunosuppression induction on day 8, background chemiluminescence (CL) intensity showed non-significant decreases in both the control and tannic acid groups (Figure S6A). Activated CL intensity and total ROS production decreased in experimental groups while remaining unchanged in controls.

In the Aronia tannin fraction group, spontaneous CL response time accelerated 3.9-fold compared to pre-CP values and was 3.26-fold faster than in controls on day 8 (*p* < 0.05). Meanwhile, activated CL response time remained unchanged.

The activation index increased in the control group on day 7 post-CP administration and was 2.27-fold higher than in the Aronia tannin fraction group (Figure 9C) (*p* < 0.05). After another 7 days, the activation index decreased 8.25-fold relative to day 14 values (*p* < 0.05).

On day 14 post-cytostatic administration, background CL intensity increased 14.6-fold in controls, 19.2-fold in the Aronia tannin group, and 9.2-fold in the tannic acid group (*p* < 0.05). Meanwhile, activated CL intensity showed no significant changes.

The background CL curve slope increased 4.4-fold in controls compared to day 8 values (*p* < 0.05), while the activated CL curve slope increased 49-fold. Conversely, in the Aronia tannin group, the activated CL curve slope decreased 1.24-fold (*p* < 0.05), indicating a more balanced cellular response without excessive load, particularly given the unchanged T_max_.

On day 21, spontaneous CL response time accelerated 4.45-fold relative to day 8 values in controls (*p* < 0.05), returning to baseline levels. Notably, due to slow ROS release, T_max_ in controls was 1.5-fold longer than in the Aronia tannin group (*p* < 0.05). Activated CL response time showed no significant changes.

Spontaneous CL AUC increased 10.7-fold and 19.3-fold relative to baseline in controls and the tannic acid group, respectively, on day 21 (*p* < 0.05). Activated CL AUC similarly increased 7-fold and 14.5-fold in these groups (*p* < 0.05). In the Aronia tannin group, AUC values were 5.2-fold higher than baseline but 3-fold lower than day 7 values (*p* < 0.05).

The activation index decreased 1.83-fold relative to day 8 values in the tannic acid group (*p* < 0.05).

## 3. Discussion

This study optimized a method for the extraction and purification of tannins from berries of the *Aronia melanocarpa* cultivar ‘Chernookaya’. A highly purified tannin fraction was obtained, suggesting that the observed biological activity is likely primarily attributable to the concentrated tannins.

A comprehensive assessment of antioxidant activity using three distinct methods (DPPH, TRAP, TAR) demonstrated that the efficacy of the antioxidant is dependent on the mechanism of action of the test system. The DPPH assay is primarily sensitive to direct Hydrogen Atom Transfer (HAT) or single Electron Transfer (ET) reactions [20]. The solubility, steric hindrance, and redox potential of quercetin may render it less effective in the DPPH assay compared to ascorbate [21]. Tannins are excellent hydrogen atom donors, which accounts for their high activity in the DPPH test [22,23]. They effectively terminate chain reactions by donating a hydrogen atom to the radical.

However, in more complex systems, such as a model of lipid peroxidation, their efficacy is reduced. This decrease is likely due to the absence of synergistic effects with other polyphenols and is determined by their ability to interact with membrane structures [24].

The higher activity of tannic acid in the DPPH assay may be attributed to its high purity and optimal molecular weight. However, its weak activity in the TRAP test at low concentrations could be due to its specific structure, which is less effective at chelating metal ions compared to Aronia tannins. In addition, the sharp decrease in activity upon dilution is a characteristic feature of polymeric antioxidants [24,25]. In the chemiluminescence (CL) assay, the Aronia tannin extract demonstrated superior efficacy compared to the other compounds. This enhanced performance may be attributed to its complex natural composition, which includes various phenolic acids and flavonoids capable of acting synergistically in complex biological systems.

Several mechanisms may explain the membrane-protective effects of tannins against free radical-induced hemolysis. First, the inherent antioxidant activity of tannins plays a key role in their protective properties [26]. By reducing the oxidative stress associated with radical species, tannins help prevent the degradation of cellular membranes. Second, tannins can chelate metal ions, such as iron, thereby inhibiting Fenton reaction activity [27]. Third, tannins can interact directly with erythrocyte membranes, modulating their fluidity and stability. The polyphenolic structures of tannins form hydrogen bonds with the polar heads of phospholipids and with membrane proteins, which increases membrane rigidity and reduces its permeability to ions and water. This reinforcement makes the membrane more resistant to osmotic stress [28,29]. This same complex-forming ability helps to “shield” the membrane from reactive oxygen species (ROS) attack.

Regarding ascorbate, in addition to its well-known direct radical-scavenging function, its role as a low-molecular-weight compound may involve the regulation of osmotic balance and cellular hydration, as well as the protection of thiol groups (-SH) in membrane proteins from oxidation, thereby helping to maintain cytoskeletal integrity [30]. This versatility makes it exceptionally effective in this model system. In contrast, Trolox effectively scavenges radicals in the aqueous phase (as confirmed by the DPPH data), but its efficacy is limited in protecting lipid membranes. As a hydrophilic compound, it incorporates poorly into the hydrophobic lipid bilayer, where lipid peroxidation primarily occurs. This supports the thesis that lipophilicity is a critical property for membrane-protective agents [18].

The results regarding the membrane-protective activity of quercetin may be explained by the low bioavailability of this flavonoid [31]. The experimental data indicate that the hemolytic safety profile of quercetin is suboptimal, as its beneficial effects are observed only at high concentrations. Quercetin, while known for its antioxidant properties, can exhibit pro-oxidant activity under certain conditions, particularly at low concentrations [32]. This implies that instead of protecting cells from oxidative stress, it may promote the formation of reactive oxygen species (ROS), damaging cellular structures such as erythrocyte membranes and leading to hemolysis.

Cytotoxicity analysis of Aronia tannins revealed a concentration-dependent effect: at low concentrations, a protective effect predominates, mediated by the activation of the Nrf2 pathway, while at high concentrations, pro-oxidant and hepatotoxic effects are observed. Thus, the pharmacology of tannins is determined by dose, exposure time, and metabolic context.

This comprehensive study demonstrated that both types of investigated tannins—tannic acid and the Aronia tannin fraction—effectively reduce the intensity of cyclophosphamide-induced lipid peroxidation, as confirmed by the dynamics of MDA level reduction [33]. However, significant differences were identified in the kinetics and mechanisms of their protective action. Tannic acid demonstrates a rapid protective effect, most effectively preventing the acute phase of oxidative damage on the 8th day after cyclophosphamide administration. This effect is likely due to its direct radical-scavenging activity and ability to chelate iron ions [34]. Furthermore, tannic acid may inhibit cytochrome P450 [15], thereby reducing the activation of cyclophosphamide and the formation of its toxic metabolites.

In contrast, Aronia tannins exhibit a delayed but more potent and sustained antioxidant effect, reaching maximum efficacy by days 14–21 of the experiment. The unique complex of oligomeric proanthocyanidins in Aronia [5] provides not only direct antioxidant action but also activation of endogenous defense systems via the Nrf2/ARE pathway [35]. This activation leads to the induction of glutathione synthesis, heme oxygenase-1, and other components of the antioxidant defense system.

Based on the hematological analysis, it can be concluded that the administration of CP induces the expected myelosuppression, manifested as leukopenia, lymphopenia, and thrombocytopenia. Conversely, the relative proportion of monocytes and neutrophilic granulocytes increased in response to CP. This may be attributed to the shorter life cycle of these cells, potentially conferring greater resistance to the immediate cytotoxic effects of CP.

The effects of tannic acid and the Aronia tannin fraction on hematopoiesis were evident through their immunomodulatory and stimulatory actions on myelopoiesis, particularly during the recovery phase from immunosuppression. The increased monocyte count in the group receiving the Aronia tannin fraction prophylactically is consistent with data indicating that Aronia polyphenols can stimulate monocytopoiesis and activate the monocyte-macrophage system [36,37]. A gradual recovery of all hematological parameters was observed on days 14 and 21. In the tannic acid group, a pronounced stimulation of the granulocytic lineage was noted, as evidenced by a significant and progressive increase in granulocyte counts on days 14 and 21. This suggests that tannic acid may accelerate myelopoiesis and promote granulocyte recovery. The more moderate granulocytic response in the Aronia tannin group compared to the control and tannic acid groups indicates a selective action of the complex natural tannins, which appears to prevent an excessive neutrophil response [38,39] and exhibits lineage-specific effects.

CP administration caused a moderate decrease in erythrocyte parameters, indicating suppressed erythropoiesis. By day 21, a more pronounced recovery of erythroid indices was observed in the control group, which may be a consequence of a strong compensatory reaction. The higher Mean Corpuscular Hemoglobin Concentration (MCHC) in the experimental groups on day 21 suggests improved hemoglobinization of erythrocytes, potentially due to antioxidant protection of erythroid precursors and the preservation of their functional activity [40].

The smaller reduction in platelet count following CP administration in the tannic acid group (1.12-fold) and the Aronia tannin group (1.32-fold) compared to the control group (1.76-fold) demonstrates the protective effect of the investigated compounds. A 17% increase in the Mean Platelet Volume (MPV) in the Aronia tannin fraction group, exceeding control values by 13%, indicates an active release of young platelet forms into the peripheral blood. This is a marker of recovering thrombopoiesis and is consistent with data on the stimulatory effect of polyphenols on megakaryocytic differentiation [41]. These findings point to a potential hem-stimulating effect of the Aronia tannin fraction, promoting the recovery of hematopoiesis after cyclophosphamide-induced myelosuppression. This effect is manifested in the accelerated recovery of both the granulocytic and megakaryocytic (platelet) lineages.

Analysis of the phagocytic activity data demonstrates that Aronia tannins exert a complex regulatory effect on the phagocytic system under conditions of cyclophosphamide-induced immunosuppression. The observed effects align with current understanding of the immunomodulatory properties of polyphenolic compounds and their ability to influence the functional activity of myeloid cells.

The control group exhibited a characteristic response to CP administration: an initial hyperactivation of the remaining neutrophils, followed by their functional exhaustion, which corresponds with known data on the myelosuppressive action of alkylating agents [42]. A compensatory increase in monocytic phagocytosis was observed; however, it was accompanied by impaired phagocytic completion, indicating defects in bactericidal mechanisms.

A fundamentally different response pattern was identified in the groups receiving tannins. Of particular interest is the ability of the Aronia tannin fraction to induce pronounced activation of the monocytic compartment while simultaneously modulating neutrophilic activity. This selective action may be associated with the ability of polyphenols to interact with Toll-like receptors on monocytes/macrophages, as described in studies on the immunomodulatory properties of plant tannins [43,44].

The temporary suppression of neutrophilic phagocytosis by tannins is likely mediated through the inhibition of NADPH oxidase and a reduction in ROS-dependent processes, while simultaneously powerfully activating the monocytic arm of immunity. This selective effect may be associated with the high content of proanthocyanidins in the Aronia tannin fraction, which interact with monocyte receptors (TLR4/CD14), promoting their polarization towards a pro-inflammatory phenotype [45].

An important aspect is the preservation of a high phagocytic completion rate in the experimental groups, indicating the ability of tannins to support not only the uptake but also the intracellular destruction of bacterial particles. This effect may be attributed to the antioxidant properties of polyphenols, which protect phagocytic cells from the oxidative stress induced by both cyclophosphamide and their own bactericidal systems [46].

The observed dynamics of the recovery processes are particularly noteworthy. In the groups receiving tannins, especially the Aronia tannin fraction, a faster and more complete restoration of phagocytic activity was observed compared to the control. These data correlate with studies demonstrating the ability of polyphenols to stimulate hematopoiesis and accelerate reparative processes in the hematopoietic system following chemotherapy [47].

The obtained results, specifically the accelerated response time of spontaneous chemiluminescence (CL), indicate the ability of the tannin fraction to activate signaling pathways, thereby increasing the readiness of neutrophils to generate ROS even at rest and enhancing the assembly of NADPH oxidase after CP-induced suppression. The moderate rate of CL increase after stimulation in the group receiving the tannin fraction confirms the protective action of tannins in reducing oxidative stress and restoring energy metabolism [48].

The prolonged but less intense oxidative response in the control group on day 14 following pharmacologically induced immunosuppression could be a consequence of partial exhaustion of oxidative metabolism, as well as the activation of alternative ROS generation pathways. The high initial rate of the CL curve may indicate rapid initial activation of signaling pathways, but with subsequent exhaustion (e.g., due to a deficit of substrates for NADPH oxidase) [49].

In the group receiving tannic acid, this effect was less pronounced. Regarding the group receiving the Aronia tannin fraction, the slight increase in the Area Under the Curve (AUC) coupled with a low peak and a slow rate of increase suggests suppression of the oxidative burst. The cause may be inhibition of NADPH oxidase, scavenging of ROS, or reduced neutrophil activation, as well as a slowing of metabolic processes, which has been reported previously [50,51,52,53]. These data are consistent with the antioxidant and anti-inflammatory action of tannins, aimed at inhibiting excessive neutrophil activation, thereby preventing tissue damage while maintaining an adequate immune response [54].

Thus, the Aronia tannin fraction possesses a unique complex of biological properties that favorably distinguishes it from both synthetic antioxidants and other natural compounds.

## 4. Materials and Methods

### 4.1. Sampling

Biologically mature fruits of *Aronia melanocarpa* cultivars ‘Chernaya Zhemchuzhina’ (Black Pearl) and ‘Chernookaya’ were used in this study. Ripe Aronia fruits in the form of umbellate inflorescences from both cultivars were collected at the “Shuyskie Yagody” farm (Ivanovo Oblast, Russia) during the harvest season in September 2023.

Berries were randomly sampled from ten shrubs per cultivar, with a quantity of 0.5 kg collected per plant. The shrubs were seven years old at the time of harvest for both cultivars. Berries of the ‘Chernaya Zhemchuzhina’ cultivar had a sour-sweet, slightly astringent taste, a dark blue color with a waxy bloom, bright red flesh, and a spherical shape with a maximum diameter of 1.0 cm and an average weight of 1.4 g. Berries of the ‘Chernookaya’ cultivar had a sour-sweet, tart-astringent taste, a black color, bright red flesh, and a rounded shape with a maximum diameter of 0.8 cm and an average weight of 1.1 g.

The berries were separated from the inflorescences, rinsed with distilled water to remove dust and contaminants, patted dry with a cotton cloth, and stored in a freezer at −35 °C for subsequent analysis.

### 4.2. Chemicals and Reagents

All chemicals and reagents used were of analytical grade. Folin–Ciocalteu reagent, 2,2-diphenyl-1-picrylhydrazyl (DPPH), and Sephadex LH-20 gel filtration resin were purchased from Sigma-Aldrich (St. Louis, MO, USA). Gallic acid, tannic acid, and rutin were purchased from PhytoLab GmbH & Co. KG, Germany (Merck KGaA, Darmstadt, Germany). Glass wool was purchased from Scharlab S.L. (Barcelona, Spain).

### 4.3. Extract Preparation

The initial stage of the work involved obtaining extracts from frozen *Aronia melanocarpa* berries using the maceration method. Frozen berries of the ‘Chernaya Zhemchuzhina’ (Black Pearl) cultivar were ground to a paste-like consistency in a laboratory mill with forced cooling of the grinding chamber (KN 195 Knifetec, Foss Tecator, Hölleröd, Denmark). The maceration was performed in a flat-bottomed conical flask that had been previously purged with argon. The crushed berries were extracted with 70% ethanol at a biomass-to-solvent ratio of 1:5. The homogenates were stirred using an automatic magnetic stirrer (RCT basic, IKA, Staufen, Germany) at 450 rpm and a temperature of 75 °C for 1.5 h under a constant stream of dry argon to prevent oxidation of the active compounds.

The resulting homogenate was centrifuged to separate the coarse sediment at 13,500 rpm for 15 min at +5 °C to avoid oxidative processes (centrifuge H3-18KR, Hunan Kecheng Instrument Equipment Co., Ltd., Changsha, China). The supernatant was concentrated using a rotary evaporator (LabTex Re 100-Pro, Labtech Company, Moscow, Russia) over a water bath at a temperature of 30–32 °C and a pressure of 13.3 mbar until the solvent was completely removed. The dried extract was stored in a medical freezer at −35 °C for further analysis. The extract from frozen berries of the ‘Chernookaya’ cultivar was obtained similarly. For the quantitative determination of major groups of phytochemicals, a portion of the dry extract was dissolved in 70% ethanol.

### 4.4. Tannin Extract Preparation

The second stage of the work involved obtaining a tannin concentrate from extracts of *Aronia melanocarpa* fruits that showed high initial tannin content upon extraction with 70% ethanol. Tannin fractions from the *Aronia melanocarpa* fruit extract were obtained and characterized according to the methods described in [55,56,57,58].

Frozen and crushed berries of the *Aronia melanocarpa* cultivar ‘Chernookaya’ were extracted using single maceration with a 50% ethanol solution containing 0.22% (*w*/*v*) sodium hydroxide to create a buffered system at pH = 8.0. The extraction was performed at a biomass-to-solvent ratio of 1:5, a temperature of 75 °C, for 1.5 h. Extracts were similarly obtained using 70% acetone and 75% methanol as extracting agents. The homogenates were then centrifuged and dried as described in the first stage (Section 4.3).

### 4.5. Preparation of Tannin Fraction

The third stage involved the isolation and purification of the tannin fraction from the extract of *Aronia melanocarpa* cultivar ‘Chernookaya’, which demonstrated high initial tannin content in the second stage.

For this purpose, an extract was prepared from frozen and crushed berries of the ‘Chernookaya’ cultivar, similarly, using maceration conditions with a 50% ethanol solution and 0.22% (*w*/*v*) sodium hydroxide. The resulting homogenate was purified as described in the first stage (Section 4.2) and concentrated under vacuum (13.3 mbar) to a final concentration of 7% (*w*/*v*). The obtained extract was filtered through a Schott funnel with a 10–16 μm pore size (Class 4) glass filter at 20 mbar for additional purification and clarification.

Column chromatography was used for the purification and isolation of the tannin fraction. Sorbents such as Sepharose, Sephadex, polyamide, and Silicagel are commonly used for the separation, isolation, and purification of major phytochemical groups from plant extracts [57,59,60].

In this work, the gel filtration resin Sephadex LH-20 with a particle size distribution of 25–100 μm was used as the stationary phase for tannin isolation and purification [55].

The sorbent material was packed into a glass column (3 × 55 cm). The bottom of the column was lined with a layer of glass wool, followed by a 25 cm high layer of the gel filtration resin, and then topped with another layer of glass wool to maintain the integrity of the absorbent layer. The column was washed with the respective solvent used for extraction, after which a 7% solution of the tannin concentrate was passed through it three times until the sorbent was fully saturated.

To separate anthocyanins, 50% ethanol acidified with 1% (*v*/*v*) HCl was used. After the near-complete disappearance of the violet color and the absence of anthocyanin traces on TLC plates with the specified solvent, elution was performed using the following solvent systems: (I) water/methanol (25:75, *v*/*v*), (II) methanol/acetone (75:25, *v*/*v*), (III) acetone/water (75:25, *v*/*v*). Twenty eluted fractions of 15 mL each were collected.

Each fraction was analyzed by thin-layer chromatography (TLC) on aluminum-backed silica gel 60 F254 plates. Visualization was performed using a TCX 254/365 UV lamp at a wavelength of 254 nm. Using the same mobile phase for TLC, the distribution coefficient (Rf) was determined. The Rf values for fraction groups 3–6, 9–11, and 14–17 were similar, measuring 0.64, 0.57, and 0.43, respectively. Fractions 3–6, 9–11, and 14–17 represented mixtures with the highest tannin content and were subsequently pooled. The combined fractions were concentrated to crystalline, fine particles using a rotary evaporator at a temperature of 35–37 °C and a pressure of 0.10–0.13 mbar. The resulting dried samples were powders: fraction 3–6 was light violet, fraction 9–11 was light beige, and fraction 14–17 was light yellow. For the determination of major phytochemical groups and antioxidant activities, a portion of the dry extract was dissolved in 50% ethanol to obtain a 1% solution.

#### 4.5.1. High-Performance Liquid Chromatography (HPLC)

Identification of flavonoids, anthocyanins and sugars in the 50% ethanolic extract (with 0.22% sodium hydroxide) and in tannin fractions 9–11 was performed by High-Performance Liquid Chromatography (HPLC) using an Agilent 1260 Infinity II LC system with a Multiple Wavelength Detector (Agilent Technologies, Inc., Santa Clara, CA, USA). HPLC analysis was carried out using Zorbax Eclipse C18 (4.6 × 150 mm), Agilent Hiflex H (250 × 4.6 mm) columns and Zorbax Carbohydrate (250 × 4.6 mm, 5 µm) (Agilent Technologies, Inc., Santa Clara, CA, USA).

#### 4.5.2. Hydrophilic Interaction Liquid Chromatography (HILIC)

For flavonoid detection, elution was performed using a gradient method. The mobile phase consisted of a mixture of 0.1% formic acid solution and acetonitrile in ratios of 1:0, 1:9, and 9:1. The flow rate was 1.0 mL/min, the injection volume was 10 µL, the run time was 30 min, and the column temperature was set at 45 °C [61].

Anthocyanins were determined by hydrophilic interaction liquid chromatography (HILIC) on a reversed-phase column; elution was carried out with a solution consisting of 30% acetonitrile and 5% formic acid in distilled water. The column temperature was 40 °C, and the mobile phase flow rate was 1 mL/min. The injection volume was 15 µL [62].

Individual sugars were quantified using hydrophilic interaction liquid chromatography (HILIC) on a Zorbax Carbohydrate reversed-phase column (250 × 4.6 mm, 5 μm). Carbohydrate elution and separation were performed in isocratic mode with a mobile phase consisting of acetonitrile and water in an 82:18 ratio. The elution flow rate was 2 mL/min, the injection volume was 10 μL, the run time was 30 min, and the column temperature was maintained at 35 °C [63,64].

#### 4.5.3. Fourier-Transform Infrared (FT-IR) Spectra

Fourier-transform infrared (FT-IR) spectra of the samples were recorded on a Tensor 27 spectrometer (Bruker, Ettlingen, Germany) using KBr pellets in the wavenumber range of 4000 to 400 cm^−1^. For this purpose, 1.5 mg of the dried tannin fraction 9–11 sample was thoroughly ground in a mortar with 150 mg of potassium bromide, placed in a press mold, and compressed under a pressure of 7–10 tons. This resulted in a uniformly transparent pellet (pellet diameter 10 mm, thickness 1 mm). The FT-IR spectrum of tannic acid was recorded as a standard following the same procedure described above for the tannin fraction. The OPUS 7/2012 software package was used for measurement, transformation, and evaluation of the obtained spectral data [65].

### 4.6. Quantification of the Main Phytochemicals

#### 4.6.1. Total Phenolic Compounds Content

The total phenolic content (TPC) was determined spectrophotometrically using the Folin–Ciocalteu method with some modifications. Briefly, a portion of the dry extract was dissolved in 70% ethanol. Subsequently, 0.5 mL of the extract aliquot was mixed with 0.5 mL of Folin–Ciocalteu reagent and kept at 24–26 °C for 5 min. Then, 2 mL of a 7.5% sodium carbonate solution was added. The volume was adjusted to 8 mL with water, and the solution was incubated in the dark for 60 min. The absorbance was measured at λ_max_ = 725 nm using a stationary scanning spectrophotometer (UV/VIS Spectrometer T7DS, China). Gallic acid was used as the standard for the calibration curve at various concentrations ranging from 0 to 200 µg/mL. The results were expressed as milligrams of gallic acid equivalents per gram of dry extract (mg GAE/g dry extract) [66].

#### 4.6.2. Total Flavonoid Content

The total flavonoid content was determined using a scanning spectrophotometer according to the Stanković method with some modifications. To an aliquot of the re-dissolved extract, 0.1 mL of 10% aluminum chloride, 0.1 mL of 1 M potassium acetate, 1.5 mL of 80% methanol, and distilled water were added. The solution was then incubated at 25 °C for 30 min. A reference solution was prepared in the same manner, except that a rutin solution of known concentration was used instead of the extract. Absorbance was measured at λ_max_ = 415 nm using a cuvette with a 10 mm optical path length. The flavonoid concentration was expressed as milligrams of rutin equivalents per gram of dry extract (mg RE/g DE) [67].

#### 4.6.3. Total Anthocyanin Content

The anthocyanin content was determined using the spectrophotometric pH differential method. For each measurement, two test solutions were prepared: (a) 1 mL of extract + 4 mL of potassium chloride/hydrochloric acid buffer (pH 1.0), and (b) 1 mL of extract + 4 mL of sodium acetate/hydrochloric acid buffer (pH 4.5). Absorbance was measured at 520 nm and 700 nm, respectively, using a cuvette with a 10 mm optical path length. The total anthocyanin content was expressed as milligrams of cyanidin-3-glucoside equivalents per gram of dry extract (mg CGE/g DE) [68].

#### 4.6.4. Total Tannin Content

The total tannin content was determined using the Folin–Ciocalteu method with some modifications. Briefly, 0.1 mL of extract was added to a 10 mL volumetric flask containing 7.5 mL of distilled water, 0.5 mL of Folin–Ciocalteu phenol reagent, and 1 mL of a 35% sodium carbonate solution, and then diluted to volume with water. The mixture was shaken and incubated at 24–26 °C for 30 min. Absorbance was measured at λ_max_ = 700 nm using a cuvette with a 10 mm optical path length. Tannic acid was used as the standard for the calibration curve in the range of 0 to 100 μg/mL (20, 40, 60, 80, 100 μg/mL). The tannin content was expressed as milligrams of tannic acid equivalents per gram of dry extract (mg TAE/g DE) [69].

#### 4.6.5. Sugar Content

The total soluble sugar content was determined spectrophotometrically using 3,4-dimethylphenol as the reagent. Stock sugar solutions of glucose, fructose, lactose, and sucrose were prepared by dissolving 100 mg of the sugar in 25 mL of water in 100 mL volumetric flasks and diluting to the mark with 0.25% benzoic acid. Working standards (15 mg/mL) were prepared by dilution. A 0.2% solution of 3,4-dimethylphenol was prepared in 50% ethanol. A sample aliquot containing 15–150 μg of sugar was placed in a 25 mL calibrated and graduated tube. The solution was evaporated on a water bath to 1 mL, then mixed with 1 mL of the 3,4-dimethylphenol solution. Then, 72% sulfuric acid was added dropwise. The absorbance of the glucose, fructose, and lactose solutions was determined at 510 nm, and that of sucrose at 520 nm, using a cuvette with a 10 mm optical path length, with a single measurement in scanning mode within the 460–570 nm range. The solution for determining sugars in the extract was prepared by dissolution in 50% ethanol. The total soluble sugar content was calculated as the sum of reducing and non-reducing sugars and expressed as mg per gram of dry extract (mg/g DE) [70].

### 4.7. Determination of Antioxidant Activity

The antioxidant activity of Aronia fruit extracts was investigated using chemiluminescent and colorimetric methods, which determine the ability of substances to interact with free peroxyl AAPH radicals and DPPH radicals, respectively. Reagents for the AAPH method were obtained from Sigma-Aldrich, USA, and for the DPPH method from Alfa Aesar, Ward Hill, MA, USA.

#### 4.7.1. Peroxyl AAPH Assay

The chemiluminescent AAPH (2.2′-azobis (2-amidinopropane) hydrochloride) method is described by Krasowska [71] and was adapted for the Lum-1200 luminometer (LLC Disoft, Moscow, Russia) [72]. Results were processed on a personal computer using PowerGraph 3.3.12 Professional and OriginLab Pro 9.5 software. Lissi et al. [73] described two approaches to measuring total antioxidant capacity, considering the specific features of the kinetic curves: the TRAP (Total Radical-trapping Antioxidant Parameter) method and the TAR (Total Antioxidant Reactivity) method. It is considered that TRAP reflects the quantity of antioxidants in the system, while TAR reflects its activity, i.e., the rate of interaction between the antioxidants and radicals.

##### Total Radical-Trapping Antioxidant Parameter (TRAP) Method

The TRAP method is based on measuring the latent period τ of chemiluminescence (CL) and can be used to determine antioxidants such as trolox or ascorbic acid: they are characterized by a high value of the reaction rate constant with radicals and, for this reason, can be called strong antioxidants [74]. During the latent period, their complete oxidation occurs.

##### Total Antioxidant Reactivity (TAR) Method

The TAR method determined the degree of CL intensity quenching, q, at the plateau or at the maximum of the CL curve. This method is employed when the system contains predominantly weak antioxidants with low rate constants for radical interaction—significantly lower compared to the rate constant of the luminol reaction [74].

#### 4.7.2. DPPH Assay

The DPPH (2,2-diphenyl-1-picrylhydrazyl) assay was performed according to the method of Brand-Williams et al. [75] with some modifications. A stock solution was prepared by dissolving 24 mg of DPPH in 100 mL of ethanol and then stored at −20 °C. A working solution was obtained by mixing 10 mL of the stock solution with 45 mL of ethanol to achieve an absorbance of 1.1 ± 0.02 units at 515 nm using a spectrophotometer. Fruit extracts (200 μL) were incubated with 800 μL of the DPPH solution for 30 min in the dark. Absorbance was then measured at 517 nm. The antiradical activity was determined as the amount of antioxidant required to reduce the initial DPPH concentration by 50% (effective concentration (EC_50_) = [AO (mol/L)/DPPH (mol/L)]).

### 4.8. Investigation of the Protective Activity of Extracts in a Model of Osmotic and Peroxide-Induced Hemolysis of Rat Erythrocytes in In Vitro Tests

The membrane-stabilizing activity of the investigated compounds was assessed using methods that induce osmotic and peroxide damage to rat blood erythrocyte membranes [76], adapted for the BioTek Epoch microplate reader (USA) [77]. To model osmotic hemolysis, a hypotonic 0.3% sodium chloride solution was added to the erythrocyte suspension. To model free radical damage, Fenton’s reagent—a mixture of 0.01 mg/mL iron (II) sulfate heptahydrate and 0.2 mg/mL hydrogen peroxide—was used. The investigated tannin samples were added to the model systems at concentrations ranging from 0.2 to 10 mg/mL.

The degree of hemolysis was assessed by measuring the change in absorbance of the supernatants at a wavelength of 543 nm. The membrane-stabilizing effect of the extracts was evaluated as the percentage of hemolysis inhibition (I), calculated using Formula (1):

(1) $$I\;\left(\%\right)=\frac{\left(D_{exp.}-D_{contr.}\right)}{D_{contr.}}\times 100,$$

where I (%) is the percentage of hemolysis inhibition; D_exp_ is the absorbance of the experimental sample; D_contr_ is the absorbance of the control sample.

### 4.9. In Vitro Experiments on the Chang Liver Human Hepatocyte Cell Line

In vitro experiments were conducted using the Chang Liver cell line, obtained from the Russian Collection of Cell Cultures at the D.I. Ivanovsky Institute of Virology (Moscow, Russia). The Chang Liver hepatocyte-like cell line was selected for the study. It is known that the Chang Liver cell culture was initially derived from normal human liver cells but was subsequently contaminated with the HeLa cervical cancer cell line.

Cells were cultured in culture flasks according to the cell line specification, in Eagle-MEM medium («PanEco», Moscow, Russia) supplemented with 10% fetal bovine serum (FBS) («PanEco», Russia), 1% essential amino acids, and the antibiotic gentamicin at 50 µg/mL («PanEco», Russia). Cultivation took place in an incubator («Thermo Fisher Scientific», Waltham, MA, USA) at 5% CO_2_ and a temperature of +37 °C [78]. For experiments, cells were detached using a mixture of trypsin («PanEco», Russia) and versene («PanEco», Russia) in a 1:3 ratio. Culture flasks were rinsed twice with this mixture and then placed in a thermostat at +37 °C for 5 min to accelerate cell detachment. Subsequently, cells were washed off the flask surface with growth medium, and cell suspensions of the required concentration for specific experiments were prepared.

To study the cytotoxicity of the new compounds, cells were detached from the culture flask as described above, and a cell suspension with a concentration of 1.5 × 10^5^ cells/mL was prepared. The suspension was then aliquoted (200 µL per well) into a 96-well plate and incubated for 24 h in a CO_2_ incubator. After 24 h, the test compounds, diluted in complete growth medium, were added to the wells in triplicate and incubated for another 24 h in the CO_2_ incubator. A reference control, where no test compounds were added to the growth medium during cell cultivation, was included.

Cell viability was determined using the MTT assay. On the third day of the experiment, 10 µL of the dye solution (5 mg of 3-(4,5-dimethylthiazol-2-yl)-2,5-diphenyl-2H-tetrazolium bromide (MTT) per 1 mL of PBS buffer) was added to the cells to achieve a final concentration of 0.5 mg/mL, followed by incubation at 37 °C and 5% CO_2_ for 3 h. The samples were then washed with phosphate-buffered saline (PBS) to remove non-adherent cells, and the formed formazan was eluted using dimethyl sulfoxide (150 µL per well, 20 min at room temperature). The formazan concentration was determined spectrophotometrically using an Epoch microplate spectrophotometer («BioTek», Winooski, Vermont, USA) by measuring the absorbance at a wavelength of 540 nm against a reference wavelength of 620 nm. Relative cell viability was calculated using the following Equation (2):

(2) $$Relative\;Viability\;\left(\%\right)=\frac{{OD}_{test}-{OD}_{background}}{{OD}_{control}-{OD}_{background}}\times 100\%,$$

where OD_test_, OD_background_, and OD_control_ represent the absorbance of the test sample, background, and control, respectively.

The half-maximal inhibitory concentration (IC_50_) was subsequently determined. IC_50_ represents the concentration at which a substance exerts half of its maximum inhibitory effect. The IC_50_ values were calculated using the statistical tool available at https://www.aatbio.com/tools/ic50-calculator, accessed on 23 September 2025, applying a four-parameter logistic regression model. This model is typically resolved as a sigmoidal function or S-shaped curve.

### 4.10. In Vivo Experiments

All studies and protocols were approved by the Bioethics Committee of the Federal Research Center of the Kazan Scientific Center of the Russian Academy of Sciences (Protocol No. 24/1, dated 4 October 2024). The studies were conducted on male Wistar laboratory rats with induced immunosuppression achieved by administration of the cytostatic agent cyclophosphamide (CP). Against the background of induced immunodeficiency, the effects of the investigated Aronia extract substances on the animals’ immune status parameters were studied, namely, the leukocyte count and the ratio of their subpopulations in peripheral blood, as well as the functional state of immune cells (chemiluminescent and phagocytic activity).

During the experiment, over a period of 7 days, rats prophylactically received orally either water (control group), the tannin fraction of *Aronia melanocarpa* fruit extract at a dose of 50 mg/kg (based on dry weight), or a solution of tannins at concentrations equivalent to those in the tannin fraction extract. The volume of administered solutions and extracts was 0.1 mL per 100 g of body weight. Drinking water was administered to the control group to create conditions analogous to those in the experimental groups.

To induce immunodeficiency, CP was administered intraperitoneally as a single dose of 25 mg/kg to the rats on day 8 of the experiment. Oral administration of the test substances continued for the subsequent 7 days.

The first blood sample was collected on day 1, prior to the start of the experiment. These results were used as reference values. Subsequent blood analyses were performed on day 8 (i.e., 24 h after CP administration), and then on days 14 and 21 (i.e., 7 and 14 days after the administration of the extracts against the background of induced immunosuppression).

#### 4.10.1. Hematological Studies

Analysis of the quantity and subpopulations of immune cells in the rat peripheral blood was performed on an automatic hematology analyzer Mythic 18 Vet (Orphee SA, Geneva, Switzerland) using a specific reagent kit. The following parameters were determined in the rat blood: total leukocyte count (WBC), lymphocyte count (LYM), monocyte count (MON), and granulocyte count (GRA). Additionally, the red blood cell count (RBC), hemoglobin concentration (HGB), mean corpuscular hemoglobin concentration (MCHC), hematocrit (HCT), platelet count (PLT), and mean platelet volume (MPV) were measured.

#### 4.10.2. Assessment of the Functional State of Rat Blood Neutrophilic Granulocytes

The generation of ROS by neutrophils was assessed by measuring the intrinsic chemiluminescence of cells isolated from the peripheral blood of experimental rats, according to the method described by Oliferuk [79]. This was performed both without the use of a chemiluminescence activator (spontaneous activity) and in response to activation with zymosan (induced activity).

The following parameters were determined from the kinetic curve of luminol-dependent neutrophil chemiluminescence: time to reach maximum intensity (T_max_, min), maximum intensity of luminescence (I_max_, c.u.), slope of the chemiluminescence curve (Slope, kPPS × 10^3^/s), and the area under the chemiluminescence curve (AUC, c.u.) for both spontaneous chemiluminescence (AUC_1_) and zymosan-induced chemiluminescence (AUC_2_).

The enhancement of induced chemiluminescence relative to spontaneous chemiluminescence was assessed using the ratio of the areas under the chemiluminescence curves (AUC_2_/AUC_1_), thus defining the activation index (I_act_, c.u.).

Phagocytic activity was assessed by cytofluorimetry using a Guava easyCyte flow cytometer (Millipore, Darmstadt, Germany). Inactivated *E. coli* bacteria labeled with fluorescein-5-isothiocyanate (FITC) (Sigma-Aldrich, St. Louis, MO, USA) were used as the phagocytosis agent, according to the methods described by [80,81,82]. The phagocytosis reaction was carried out by incubating the leukocyte concentrate with a suspension of FITC-labeled *E. coli* bacteria for 30 and 120 min at 37 °C.

The following parameters were calculated for neutrophils and monocytes regarding FITC-labeled *E. coli*: phagocytic number (PN): the number of bacterial particles per phagocytosing neutrophil or monocyte; percentage phagocytosis (PP): the proportion of active phagocytes (those that ingested FITC-labeled bacteria) relative to the total number of monocytes or neutrophils; phagocytic index (PI): the average number of bacteria per neutrophil or monocyte; phagocytic completion index (PCI), in arbitrary units, defined as the ratio of the phagocytic number at 30 min to the phagocytic number at 120 min [81,83].

#### 4.10.3. Determination of Lipid Peroxidation Level in Rat Blood

The malondialdehyde (MDA) content in rat blood erythrocytes was determined using the thiobarbituric acid reactive substances assay, as previously described by us [84] with a slight modification of the method. The absorbance of the clear supernatant was measured at 535 nm, and the MDA concentration was calculated using 1.56 × 10^5^ M^−1^cm^−1^ as the molar extinction coefficient. The MDA results were expressed in µmol per liter of blood (µmol/L).

### 4.11. Statistical Analysis

Data were processed using Microsoft Excel 2016 and OriginPro 9.5 (OriginLab Co., Northampton, MA, USA). Data were compared using the non-parametric Kruskal–Wallis test. Exact *p*-values were calculated for pairwise comparisons between groups using the Mann–Whitney U test. Statistical analysis was performed using SPSS software, version 23.0 (Chicago, IL, USA). The obtained values are presented as mean ± standard deviation (SD). A *p*-value of less than 0.05 (*p* < 0.05) was considered statistically significant.

## 5. Conclusions

A highly purified tannin fraction was isolated from *Aronia melanocarpa*, and its compositional profile was studied using HPLC-MS and IR spectroscopy.

Based on the conducted research, it can be concluded that the Aronia tannin fraction possesses a unique complex of biological properties that favorably distinguish it from both synthetic antioxidants and other natural compounds.

The comprehensive antioxidant protective properties of the Aronia tannin fraction have been established. These are manifested by its pronounced, superior-to-quercetin ability to scavenge DPPH radicals, a membrane-protective effect exceeding the activity of reference antioxidants, and a significant reduction in the malondialdehyde level in the blood of rats subjected to cyclophosphamide-induced oxidative stress.

Furthermore, an immunomodulatory effect of the Aronia tannin fraction was revealed, which is expressed through its complex regulatory action on the phagocytic system. Specifically, it involves the selective activation of the monocytic immune compartment while simultaneously modulating neutrophilic activity. An important aspect is the preservation of a high phagocytic completion rate, indicating the ability of the tannins to support not only the uptake but also the intracellular destruction of bacterial particles.

The obtained data confirm the potential of the Aronia tannin fraction for mitigating oxidative stress and immune disorders associated with immunosuppression.

The obtained results reveal several important directions for subsequent research. Primarily, further investigation into the molecular mechanisms of action of the tannin fraction using diverse experimental models is required. These studies should aim to identify the specific molecular targets and signaling pathways mediating its immunomodulatory and antioxidant effects. A comprehensive pharmacological analysis, including the investigation of the pharmacokinetics, bioavailability, and metabolism of individual components of the tannin fraction under in vivo conditions, represents a significant scientific and practical interest. Such an analysis will enable a thorough assessment of the therapeutic potential of this natural complex. For the successful practical application of the findings, work on the standardization and optimization of the tannin fraction’s composition, as well as the development of standardized methods for its production, is necessary.

## Figures and Tables

**Table 1:** Group composition of 70% ethanolic extracts from two *Aronia melanocarpa* cultivars.

Cultivar	Total Sugar Content, mg/g DE ^1^	Total Phenolic Content, mg GAE ^2^/g DE	Total Flavonoid Content, mg Rut ^3^/g DE	Total Anthocyanin Content, mg/g DE	Total Tannin Content, mg TanA ^4^/g DE
‘Chernaya Zhemchuzhina’ (Black Pearl)	61.75	15.17	0.93	3.12	52.3
‘Chernookaya’	43.58	25.31	1.15	1.81	71.9

^1^ DE—dry extract weight; ^2^ GAE—gallic acid equivalents; ^3^ Rut—rutin equivalents; ^4^ TanA—tannic acid equivalents.

**Table 2:** Group composition of tannin concentrates from *Aronia melanocarpa* ‘Chernookaya’ cultivar obtained using different extraction solvents and the tannin fraction isolated with 50% ethanol containing 0.22% sodium hydroxide (on a dry weight basis).

Extract	Total Sugar Content, mg/g DE	Total Phenolic Content, mg GAE/g DE	Total Flavonoid Content, mg Rut/g DE	Total Anthocyanin Content, mg/g DE	Total Tannin Content, mg TanA/g DE
70% Acetone	12.25	13.78	1.27	1.52	63.57
75% Methanol	21.36	16.54	1.13	1.12	79.17
50% EtOH (pH = 8.0)	46.31	18.04	2.36	0.51	91.43
Fraction 3–6	15.6	4.73	1.04	0.130	311.5
Fraction 9–11	0.174	1.04	0.21	0.031	911.7
Fraction 14–17	7.43	1.79	0.19	-	265.8

**Table 3:** Anthocyanins identified in the extract and tannin fraction of *Aronia melanocarpa* ‘Chernookaya’ berries.

Sample	Anthocyanin Content (mg/g Dry Extract)			
	Cyanidin-3-O-galactoside	Cyanidin-3-O-glucoside	Cyanidin-3-O-arabinoside	Cyanidin-3-O-xyloside
50% EtOH (pH = 8.0)	0.236	0.072	0.113	0.083
Fraction 9–11	0.023	-	0.007	-

**Table 4:** Comparative content of individual flavonols and flavonol glycosides in the 50% ethanolic extract and tannin fraction (mg/g dry extract).

Sample	Individual Form, mg/g DE			Glycosylated Form, mg/g DE				
	Quercetin	Kaempferol	Dihydro-quercetin	Quercetin-3-O-rutinoside	Hesperetin-7-O-rutinoside	Quercetin-3-O-rhamnoside	Quercetin-3-O-galactoside	Quercetin-3-O-glucoside
50% EtOH (pH = 8.0)	1.05	0.121	0.147	0.651	0.131	0.037	0.089	0.063
Fraction 9–11	0.109	0.023	0.046	-	0.016	-	-	-

**Table 5:** Comparative content of individual sugars in the 50% ethanolic extract and tannin fraction (mg/g DE).

Sample	Total Identified Sugars, mg/g DE			
	Fructose	Glucose	Sorbitol	Sucrose
50% EtOH (pH = 8.0)	10.575	18.405	13.995	1.44
Fraction 9–11	0.043	0.072	0.054	-

**Table 6:** Results of antioxidant activity analysis in the DPPH assay. EC_50_—half maximal effective concentration. Lower EC_50_ values indicate higher antioxidant activity.

Sample	EC_50_ (mg/mL)
Aronia tannin fraction	0.029
Tannic acid	0.017
Ascorbic acid	0.010
Quercetin	0.149

## Data Availability

Data is contained within the article.

## Supplementary Materials

The following supporting information can be downloaded at: https://www.mdpi.com/article/10.3390/molecules30224338/s1, Figure S1: HPLC chromatograms of standard anthocyanin compounds. (a) Cyanidin-3-O-galactoside, retention time 23.35 min; (b) cyanidin-3-O-glucoside, retention time 24.52 min; (c) cyanidin-3-O-arabinoside, retention time 26.05 min; (d) cyanidin-3-O-xyloside, retention time 33.37 min. Figure S2: HPLC chromatograms. (a) Tannin fraction 9–11 of *A. melanocarpa*, (b) 50% ethanolic extract of chokeberry with 0.22% sodium hydroxide. Figure S3: HPLC chromatograms of standard compounds. (a) Individual flavonols; (b) glycosidic form of flavonoids; (c) 50% ethanolic extract of chokeberry with 0.22% sodium hydroxide; (d) tannin fraction 9–11 of chokeberry. Figure S4: HPLC chromatograms. (a) Standard compounds of individual sugars—fructose (1), glucose (2), sorbitol (3), sucrose (4), maltose (5); (b) 50% ethanolic extract of *A. melanocarpa* with 0.22% sodium hydroxide; (c) tannin fraction 9–11 from *A. melanocarpa*. Figure S5: Phagocytic number of granulocytes (A) and monocytes (B) in rat peripheral blood. * (*p* < 0.05)—significant difference compared to day 1 within the group. – (*p* < 0.05)—significant difference between groups. Figure S6: Values of spontaneous and zymosan-activated chemiluminescence of rat neutrophils: (A) chemiluminescence intensity (I_max_, c.u.); (B) slew rate of the chemiluminescence curve (Slope, kPPS × 10^3^/s). * *p* < 0.05, significance of differences compared to the first day of the experiment, – *p* < 0.05, significance of differences between the animal groups.

## Abbreviations

The following abbreviations are used in this manuscript:

AAPH	2.2′-azobis (2-amidinopropane) hydrochloride
AUC	area under the luminescence curve
CL	chemiluminescence
CP	cyclophosphamide
DPPH	2,2-diphenyl-1-picrylhydrazyl
I_act_	activation index
I_max_	chemiluminescence intensity
FITC	fluorescein-5-isothiocyanate
HPLC-MS	high-performance liquid chromatography–mass spectrometry
MDA	malondialdehyde
PCI	phagocytosis completion index
PI	phagocytic index
PN	phagocytic number
PP	percentage phagocytosis
ROS	reactive oxygen species
Slope	slew rate of the chemiluminescence curve
TAR	total antioxidant reactivity
TRAP	total radical-trapping antioxidant parameter
T_max_	time to peak chemiluminescence
