# Supplementary material for: Effects of Aronia melanocarpa Tannins on Oxidative Stress and Immune Dysfunction
Source: Molecules. 2025 Nov 8;30(22):4338. doi: 10.3390/molecules30224338 (PMC12654808; doi:10.3390/molecules30224338)

Figure S1: HPLC chromatograms of standard anthocyanin compounds. (**a**) Cyanidin-3-O-galactoside, retention time 23.35 min; (**b**) cyanidin-3-O-glucoside, retention time 24.52 min; (**c**) cyanidin-3-O-arabinoside, retention time 26.05 min; (**d**) cyanidin-3-O-xyloside, retention time 33.37 min.

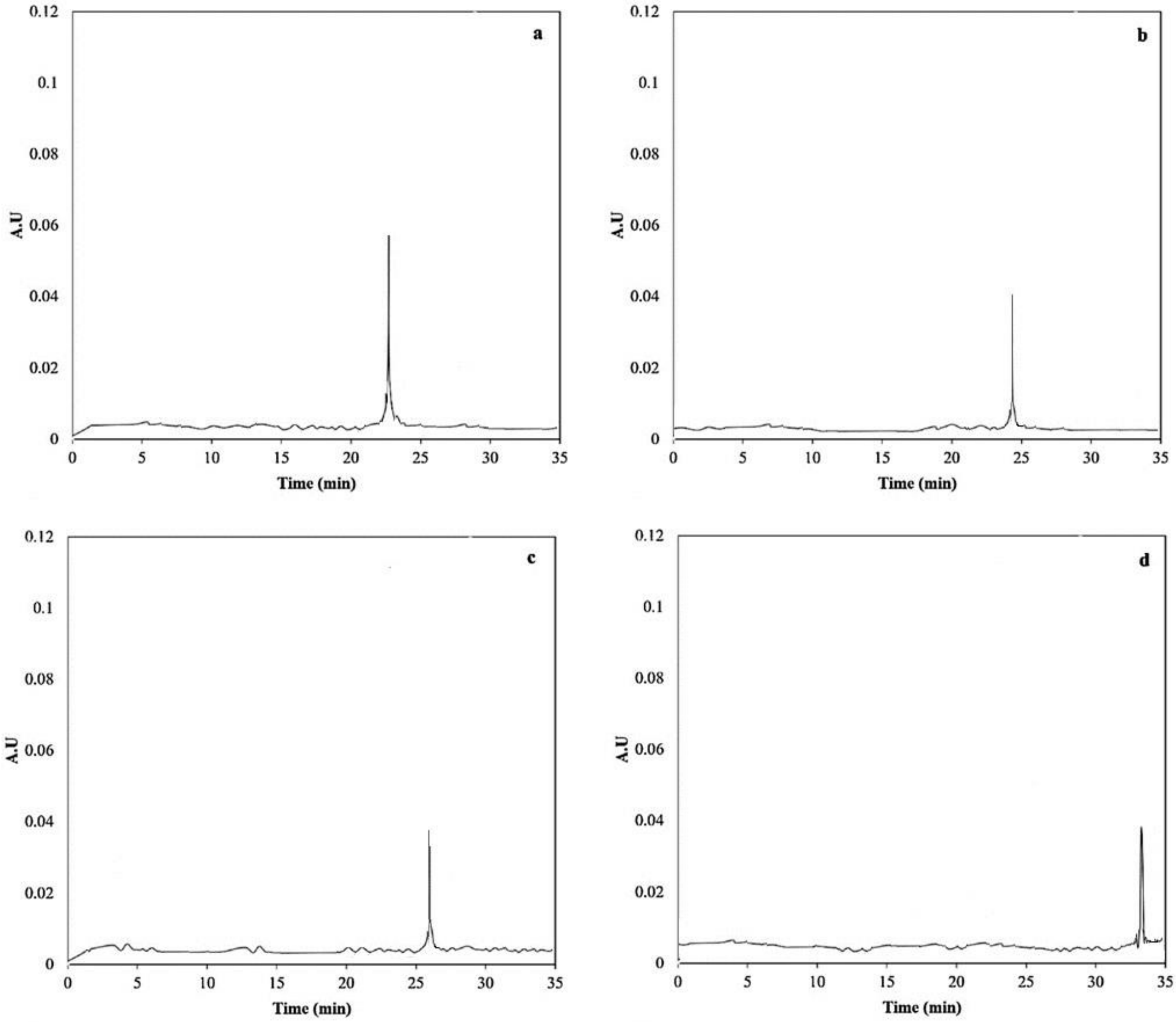

Figure S2: HPLC chromatograms. (**a**) Tannin fraction 9-11 of *A. melanocarpa*, (**b**) 50% ethanolic extract of chokeberry with 0.22% sodium hydroxide.

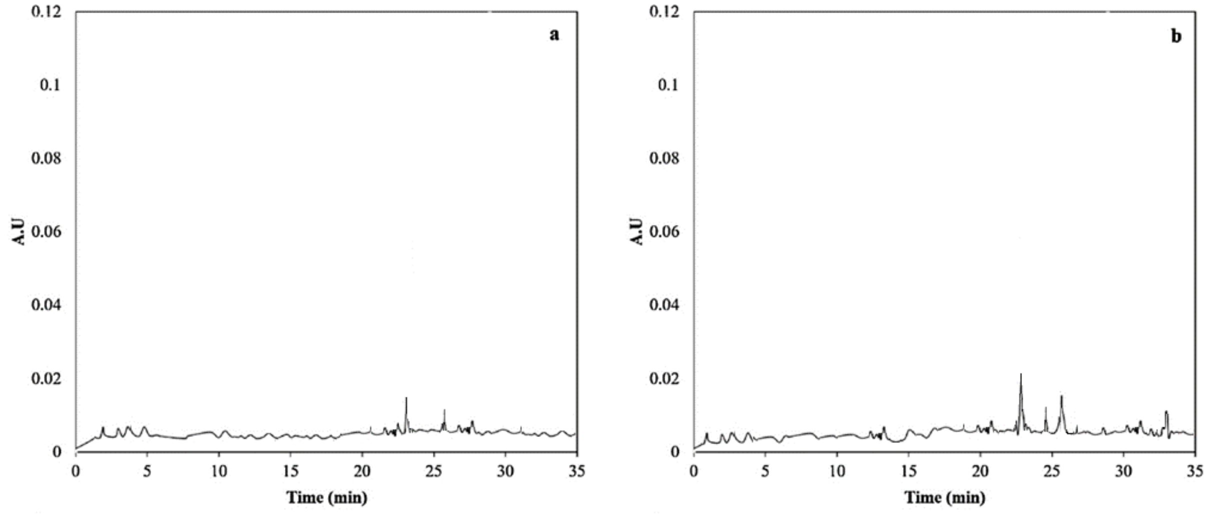

Figure S3: HPLC chromatograms of standard compounds. (**a**) Individual flavonols; (**b**) glycosidic form of flavonoids; (**c**) 50% ethanolic extract of chokeberry with 0.22% sodium hydroxide; (**d**) tannin fraction 9-11 of chokeberry.

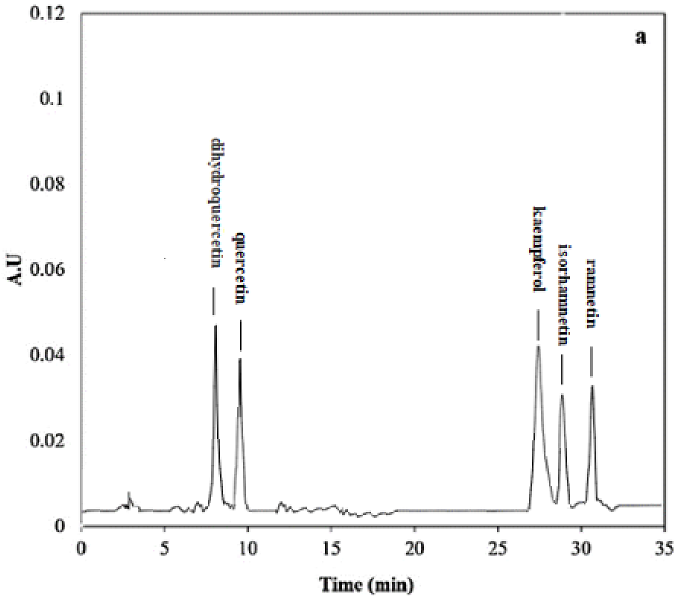

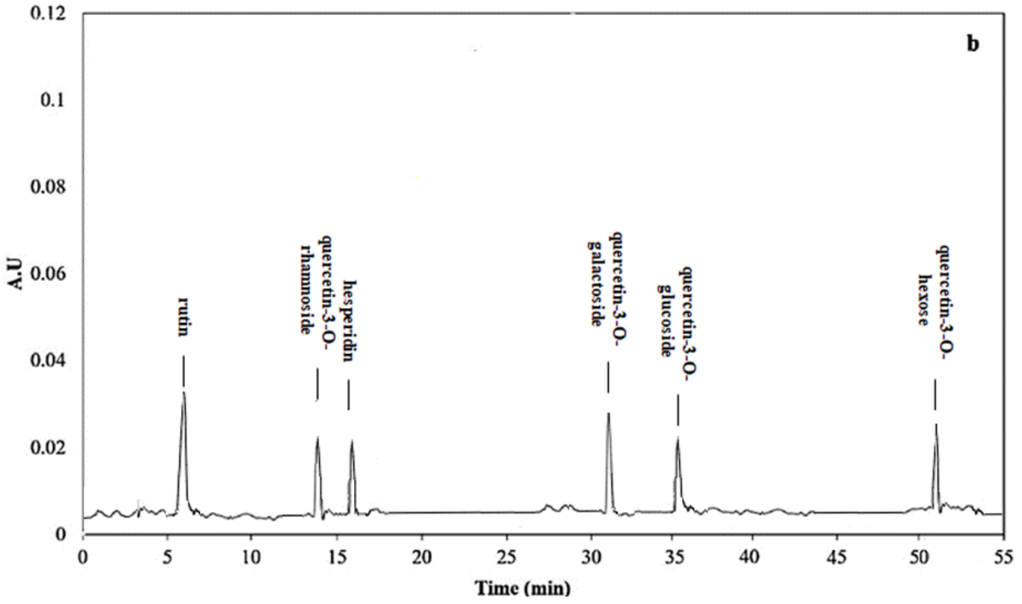

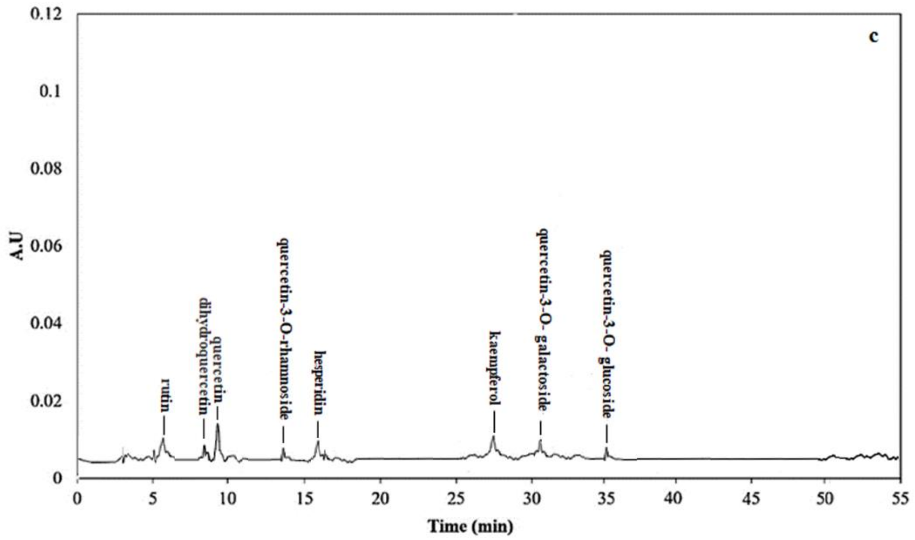

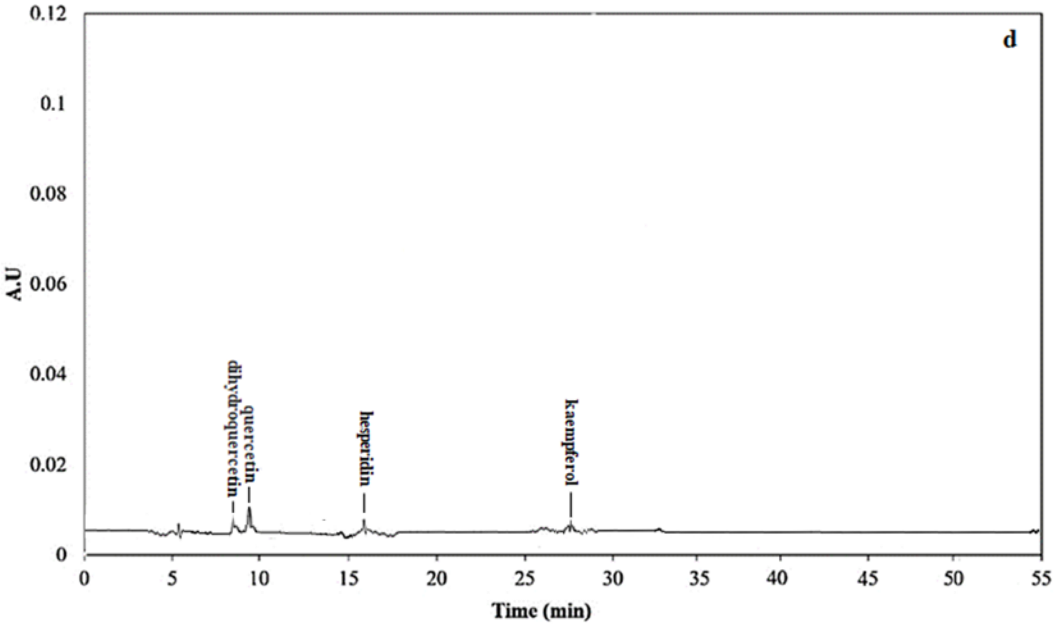

Figure S4: HPLC chromatograms. (**a**) Standard compounds of individual sugars - fructose (1), glucose (2), sorbitol (3), sucrose (4), maltose (5); (**b**) 50% ethanolic extract of *A. melanocarpa* with 0.22% sodium hydroxide; (**c**) tannin fraction 9-11 from *A. melanocarpa*.

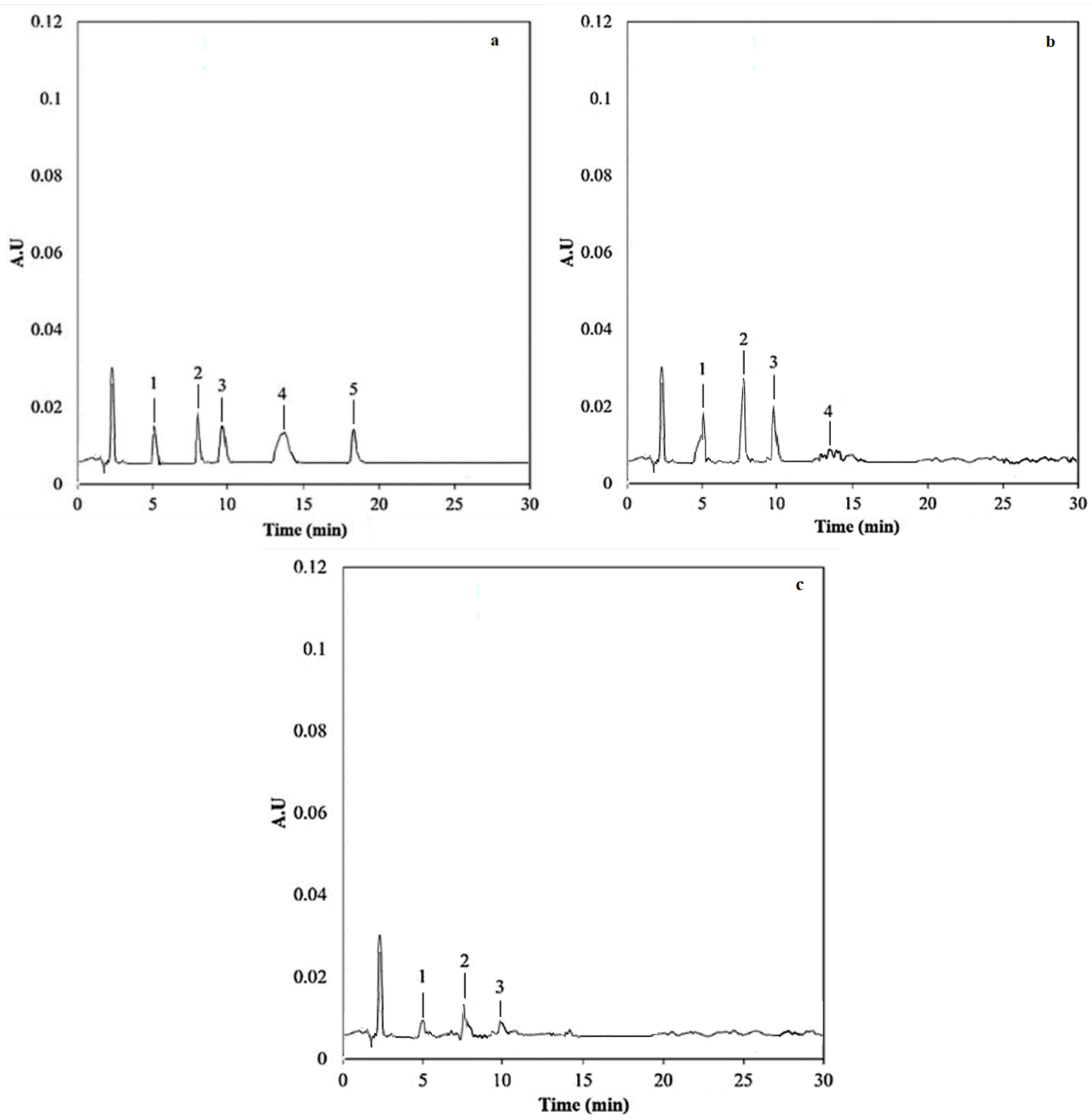
a
b
c
A.U
0.12
0.1
0.08
0.06
0.04
0.02
0
1
2
3
4
5
0
5
10
15
20
25
30
Time (min)

Figure S5: Phagocytic number of granulocytes (**A**) and monocytes (**B**) in rat peripheral blood. * ($p < 0.05$) – significant difference compared to day 1 within the group. – ($p < 0.05$) – significant difference between groups.

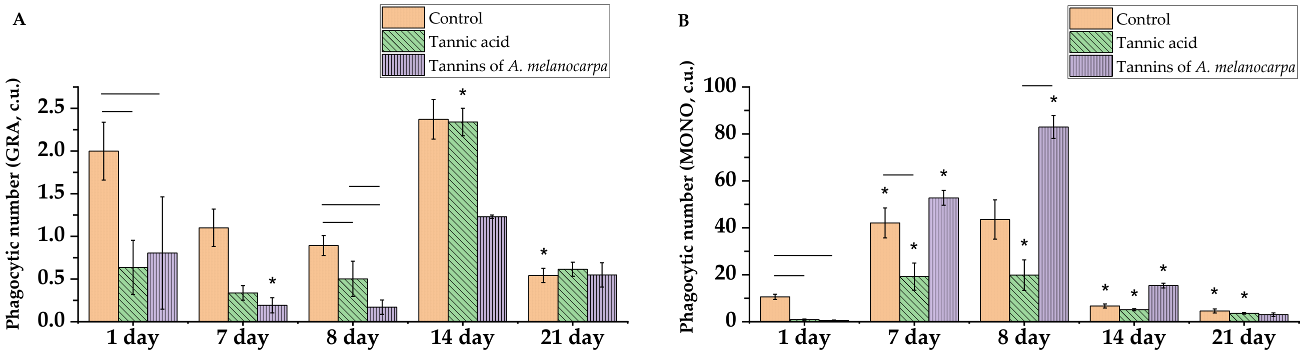

Figure S6: Values of spontaneous and zymosan-activated chemiluminescence of rat neutrophils: (**A**) chemiluminescence intensity (I_{max}, c.u.); (**B**) slew rate of the chemiluminescence curve (Slope, kPPS×10³/sec). * $p < 0.05$, significance of differences compared to the first day of the experiment, - $p < 0.05$, significance of differences between the animal groups.

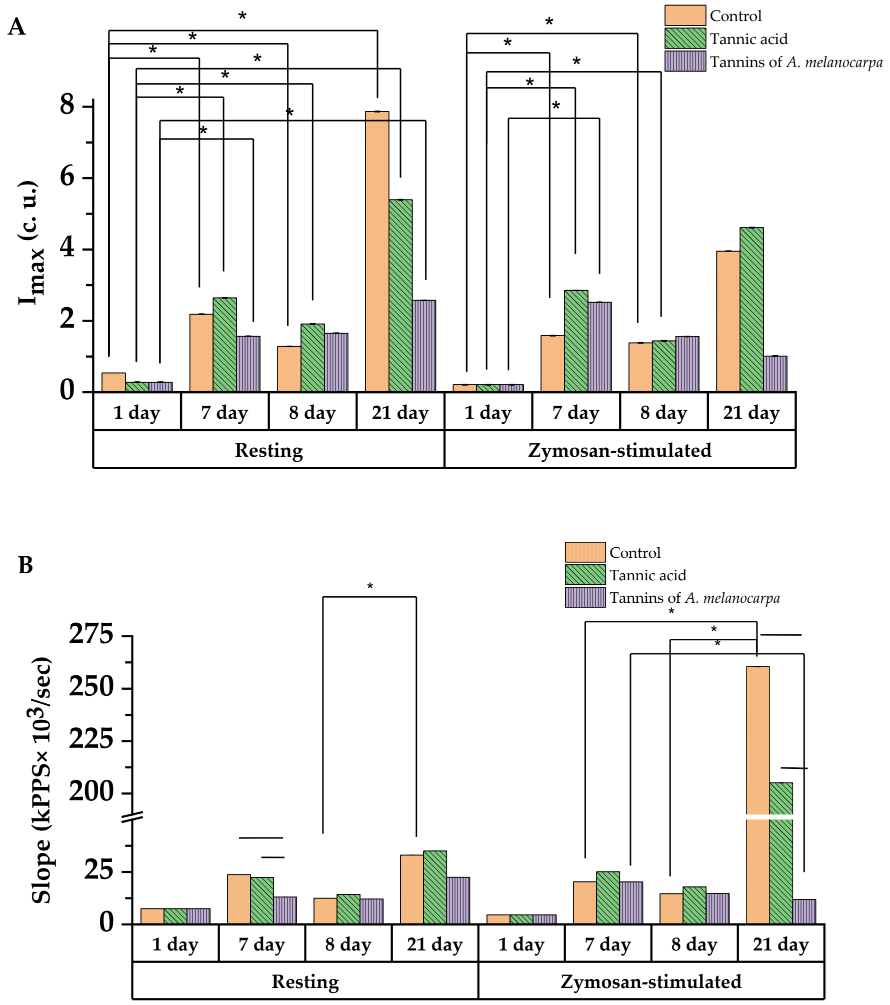

Supplement: Supplementary file 1 [file molecules-30-04338-s001.zip › molecules-3921265-supplementary.pdf]
